# Sequence-free landscape inference for directed evolution

**DOI:** 10.1371/journal.pcbi.1014713

**Published:** 2026-09-03

**Authors:** Sebastian Towers, Jessica James, Harrison Steel, Idris Kempf

**Affiliations:** Department of Engineering Science, University of Oxford, Oxford, United Kingdom; Wellcome Sanger Institute, UNITED KINGDOM OF GREAT BRITAIN AND NORTHERN IRELAND

## Abstract

Directed evolution is a method for engineering biological systems or components, such as proteins, wherein desired traits are optimised through iterative rounds of mutagenesis and selection of fit variants. The process of protein directed evolution can be envisaged as navigation over high-dimensional optimisation landscapes with numerous local maxima. The performance of any strategy in navigating such a landscape is dependent on the ruggedness of that landscape. However, this information is generally unavailable at the outset of an experiment. Here we propose **SLIDE**, **S**equence-free **L**andscape **I**nference for **D**irected **E**volution, which consists of two parts. First, SLIDE provides an estimation of landscape ruggedness from a mutating population using only population-level phenotypic data and an estimate of the mutation rate. Such ruggedness information in itself is valuable in protein design, for instance in predicting evolutionary stability. Second, SLIDE offers a framework for using the estimated ruggedness metric to identify high-performing selection strategies for directed evolution. Using theoretical *NK* landscapes and four empirical protein fitness landscapes, we demonstrate consistent *in silico* improvement upon the performance of fixed-parameter strategies, using a pipeline that could also be combined with emerging AI-based methods for driving directed evolution.

## Author summary

Directed evolution (DE) navigates high-dimensional protein fitness landscapes to find improved variants, but the success of any strategy depends on the landscape’s ruggedness, which is typically unknown and often requires genetic sequencing to derive. We present Sequence-free Landscape Inference for Directed Evolution (SLIDE), a two-step framework that (i) estimates landscape ruggedness from population-level phenotypic decay curves together with a mutation rate estimate, and (ii) uses that ruggedness estimate to choose DE control parameters that better balance exploration and exploitation. In contrast to existing ruggedness metrics, SLIDE relies on spectral analysis to link the exponential decay rate of mean fitness under random, unbiased mutations to the dominant frequency content of the landscape. This decay rate is related to the normalised Dirichlet energy of the underlying genotype graph, and the framework naturally extends to biased mutation spectra. On homogeneous landscapes, such as *NK* landscapes, ruggedness can be accurately estimated from a single starting genotype. Heterogeneous empirical landscapes instead require sampling multiple starting points, naturally distinguishing local from global ruggedness estimates. We combine SLIDE with DE strategies developed in our previous work, but the method is strategy-agnostic and can be paired with any DE workflow, including emerging AI-driven workflows. *In silico* tests show that SLIDE reliably recovers landscape ruggedness and enables more effective selection of directed evolution strategies than fixed-parameter approaches across landscapes of varying ruggedness. Although heterogeneous landscapes require fitness decay measurements from multiple starting points, these can be generated in parallel using high-throughput mutagenesis methods, such as error-prone PCR or chemical mutagenesis, thereby avoiding the need for extensive sequencing or combinatorially complete genotype–phenotype maps while remaining scalable to large screens. Beyond DE, the approach can rapidly characterise landscapes arising in genetic circuits, enzyme libraries, and ecological models, providing a way to quantify genetic stability and guide experimental or computational search.

## Introduction

Fitness landscapes map DNA or amino acid sequences to a measure of fitness such as enzyme activity, cell growth, binding affinity, or stability [1]. Fitness landscapes are characterised by multiple structural properties, such as ruggedness, epistasis, neutrality, and navigability, which have significant consequences for evolution and stability [2,3]. In this work, we focus on inferring the ruggedness of a fitness landscape, which unlocks the ability to assess the robustness of a protein to mutations and to optimise directed evolution (DE) protocols.

Various metrics have been proposed to define and quantify the ruggedness of fitness landscapes [4–8]. Although these metrics have been shown to correlate across several empirical landscapes [8], most of them require combinatorially complete genotype–phenotype maps to compute a ruggedness measure. Several of these metrics come from spectral landscape theory [9], which describes fitness landscapes using tools from Fourier analysis. In the same way that a signal can be decomposed into a sum of waves, a fitness landscape can be decomposed into a sum of landscapes of different frequencies (ruggedness). Where low-frequency terms dominate, the landscape can appear smooth, whereas where high-frequency terms dominate, the landscape can appear rugged.

**Fig 1:**
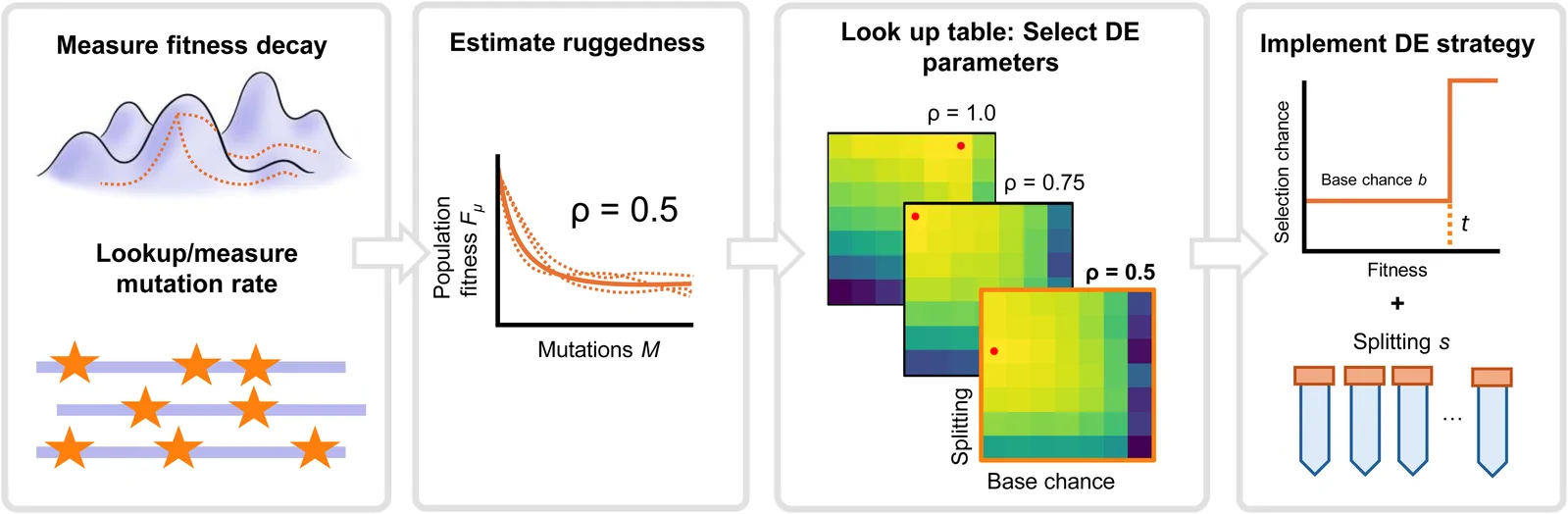
Schematic outline of a future DE pipeline. The initial phase of SLIDE involves random mutation away from one or several starting points and measurement of the resulting fitness decay curve, which, alongside a known mutation rate, can be used to estimate the fitted decay rate $\rho^{fit}$ as a measure of ruggedness. A look-up table matches analytical ρ values of *NK* landscapes to optimal DE strategy parameters. This look-up table can be used to select appropriate parameters for the measured $\rho^{fit}$. Parameters explored in this paper include base chance, which assigns a fitness-independent probability of selection, and population splitting [10].

In this paper, we develop Sequence-free Landscape Inference for Directed Evolution (SLIDE) to efficiently estimate the ruggedness of a landscape using phenotypic information alongside an estimated mutation rate (Fig 1), and we validate this pipeline *in silico*. Based on spectral landscape theory and under the assumption of random, unbiased mutations, we define a novel ruggedness metric as a normalised Dirichlet energy of the genotype graph, which can be related to the dominant Fourier terms of the landscape. We show that this metric can be estimated from the average fitness decay of a population that is randomly mutated from a starting point, unlike existing ruggedness metrics, which require combinatorially complete genotype–phenotype maps. The ruggedness metric characterises the rate at which the mean population fitness relaxes towards the landscape average; on smooth landscapes, this average fitness decays more slowly than on rugged landscapes. This phenomenon can be attributed to fitness correlations between variants on smooth landscapes, as random mutations are less likely to result in large fitness differences. Most importantly, the fitness decay curve can be acquired without large-scale sequencing data. Instead, its estimation requires only population-level fitness readouts and an estimate of the mutation rate.

In SLIDE, we combine ruggedness inference with DE. In DE, biological components such as proteins are engineered and improved through iterative rounds of selection and mutagenesis [11]. It can be seen as navigating a high-dimensional fitness landscape, aiming to guide the fitness of variants to a high-performing location on the landscape. Early DE optimisation techniques such as ProSAR used statistical models to correlate sequence with measured activity and iteratively design variants that maximise predicted fitness [12]. Since then, advances in machine learning have led to the development of more sophisticated algorithms that actively design the sequence of variants that form the next generation [13–15]. Although effective, these methods are limited by their need for high-throughput sequencing data. Without sequencing data, most DE experiments select a fixed top proportion of variants in each iteration [16–18], i.e., truncation selection (comparable to hill-climbing algorithms), which we refer to as the “baseline strategy”. This approach results in minimal landscape exploration, and is therefore prone to becoming trapped in local optima [10].

To address this limitation, several methods have been proposed for balancing landscape exploration and exploitation. Such methods include applying non-constant selection pressure [19], increasing the proportion of the population selected [20], splitting the population into sub-populations, and including a base chance of selection that is irrespective of fitness [10]. However, effective application of these methods depends on knowledge of landscape properties such as ruggedness [10]. Inferring the ruggedness beforehand is essential for selecting strategy parameters to ensure the balance of exploration and exploitation. Here, we select these parameters based on a ruggedness estimate obtained using SLIDE, in which ruggedness is estimated and the strategy parameters are selected before the DE experiment. By doing so, SLIDE increases the likelihood of reaching higher-fitness variants within a fixed number of generations, even when the decay rate is not estimated accurately.

SLIDE is strategy-agnostic and can be integrated into DE workflows both with and without sequencing by using the inferred ruggedness to tune strategy parameters that control the balance between exploration and exploitation. Although we are not aware of prior DE workflows that explicitly use a measured ruggedness scalar for this purpose, many established methods already expose suitable control knobs. For example, machine-learning-assisted DE with combinatorial libraries uses measured sequence–function data to guide library design [13], while Bayesian-optimisation methods formalise exploration–exploitation trade-offs through acquisition-function choice and aggressiveness [21]. Recombination-based workflows provide another natural target, since the optimal selection pressure can depend on whether variation is supplied primarily by mutation or recombination [22]. In this paper, SLIDE is interfaced with strategies developed in our previous work [10], controlling base chance and population-splitting parameters. We evaluate the resulting pipeline *in silico* on theoretical landscapes from the *NK* model [23] and on four empirical landscapes [2,24–26]. SLIDE automatically selects landscape-dependent strategy parameters, improving over fixed-parameter strategies such as best-performer selection on rugged landscapes and overly exploratory selection on smoother landscapes.

## Results

### Ruggedness inference from fitness decay rate

**Fig 2:**
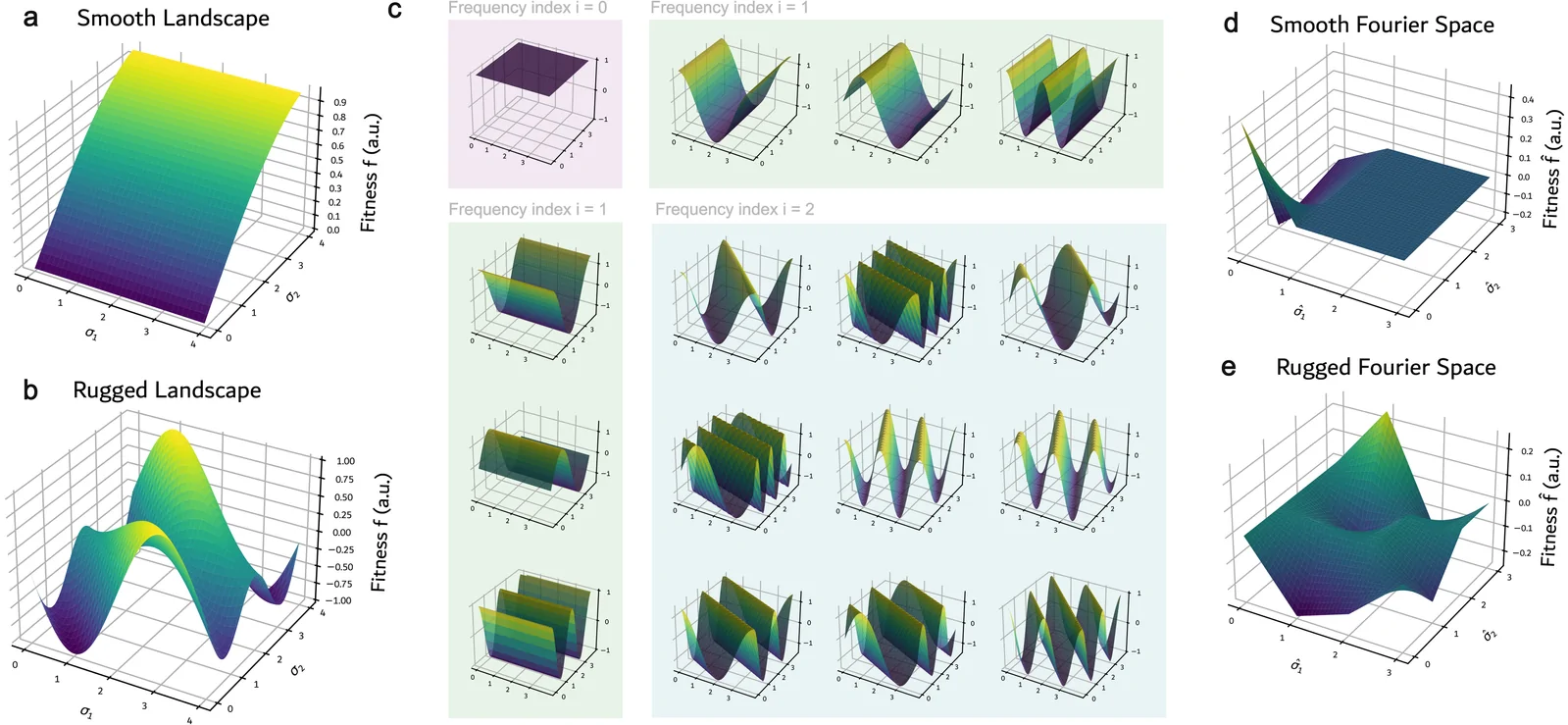
Demonstration of landscape transformations. Examples of **A:** smooth and **B:** rugged landscapes. **C:** Example Fourier basis. **D–E:** Smooth and rugged landscapes transformed into Fourier space with the example basis.

Ruggedness of a fitness landscape can be defined in terms of various mathematically distinct metrics (see [4–8]), which aim to capture how fitness changes when the landscape is traversed. Fig 2A–2B presents examples of smooth and rugged landscapes for a protein of length *N* = 2 with 16 genotypes, where $\sigma_{i}$ is used to denote the allele at sequence position i={1,2}, where each $\sigma_{i}$ takes one of *A* = 4 possible amino-acid values. Intuitively, smooth landscapes should have high fitness variants clustered together, leading to high fitness correlations and relatively few local extrema. In contrast, rugged landscapes should exhibit large fitness differences between adjacent nodes, resulting in more local extrema and lower fitness correlations.

We consider a population ν that traverses a landscape via unbiased, single-point mutations and develop a method to measure ruggedness based on spectral landscape theory [9], which decomposes landscapes into low-frequency (smooth) and high-frequency (rugged) components using a graph Fourier transform (GFT). Analogous to a standard Fourier transform, the GFT decomposes the original landscape f to express it as a mixture of basis landscapes of increasing frequency, such as those shown in Fig 2C. Here, f can be interpreted as mapping from genotype space 𝒢 to fitness, f:𝒢→ℝ, or as a vector $f\in {\mathbb{R}}^{\left|𝒢\right|}$, where |𝒢| denotes the cardinality of 𝒢. For a protein of length *N* and *A* choices of amino acids, there are $|𝒢|=A^{N}$ basis landscapes grouped into *N* + 1 frequencies $\lambda_{0},...,\lambda_{N}$. The weights of each basis landscape in f can be arranged to form a coefficient vector $\hat{f}$ in the Fourier domain, as shown in Fig 2D–2E. In the Fourier domain, genotype indices are replaced by Fourier-mode indices, and the corresponding values are the Fourier coefficients of the landscape.

Let $F_{\mu }$ denote the expected population fitness after an average of μ accumulated mutations, where the expectation is taken over mutation trajectories. We further define the expected squared population-average fitness $G_{\mu }\;\begin{array}{c} \overset{\text{def}}{=}\;{𝔼}_{\nu }[F_{\mu }^{2}] \end{array}$, where the expectation is additionally taken over all single-genotype populations. Based on the GFT, we propose to extract the scalar ruggedness metrics $\rho_{1}$ and $\rho_{2}$, associated with $F_{\mu }$ and $G_{\mu }$, respectively, as the weighted frequency averages


$$\begin{array}{cc} \rho_{1} & =\frac{1}{d}\frac{\sum_{i=0}^{N}a_{i}\lambda_{i}}{\sum_{i=0}^{N}a_{i}-a_{0}}=\frac{1}{d}\frac{f^{\text{T}}\Delta \nu }{f^{\text{T}}\nu -\bar{f}}, \end{array}$$ (1a)

$$\begin{array}{cc} \rho_{2} & =\frac{1}{d}\frac{\sum_{i=0}^{N}b_{i}\lambda_{i}}{\sum_{i=0}^{N}b_{i}-b_{0}}=\frac{1}{d}\frac{f^{\text{T}}\Delta f}{f^{\text{T}}f-A^{N}{\bar{f}}^{2}}. \end{array}$$ (1b)

where d=N(A−1) is determined by the landscape size and alphabet, $a_{i}$ denotes the population-weighted spectral coefficient at frequency index *i*, $b_{i}$ the population-independent power spectral coefficient at frequency index *i*, $\bar{f}$ the landscape average fitness, and $\lambda_{i}$ a frequency-dependent quantity determined by the genotype-graph Laplacian $\Delta =\Delta^{\text{T}}$ associated with the genotype graph induced by the unbiased mutation process (see Methods). The left-hand sides of Eqs (1a) and (1b) correspond to the centroids of the population-weighted spectrum and the population-independent power spectrum, respectively. The right-hand sides provide equivalent representations in vector form. In particular, Eq (1b) shows that the power-spectral centroid is equivalent to a normalised Dirichlet energy on the genotype graph, quantifying the average squared fitness variation between mutational neighbours. Consequently, the frequency-domain interpretation of our metrics is naturally linked to the unbiased mutation model, although we show in the Methods how the framework extends to biased mutation operators beyond the GFT. A key advantage of the metrics in Eq (1) is that they can be estimated directly from phenotypic measurements obtained from randomly mutagenised populations. In Methods, we show that for small μ, the expected population fitness $F_{\mu }$, the average squared fitness $G_{\mu }$, and the metrics from Eq (1) are related by:


$$\begin{array}{cc} F_{\mu } & =C_{1}e^{-\mu \rho_{1}}+\bar{f}+𝒪\left(\mu^{2}\right), \end{array}$$ (2a)

$$\begin{array}{cc} G_{\mu } & =C_{2}e^{-2\mu \rho_{2}}+{\bar{f}}^{2}+𝒪\left(\mu^{2}\right), \end{array}$$ (2b)

where *C*_1_ is a constant that depends on the initial population and can be negative if the initial population fitness is lower than $\bar{f}$, and *C*_2_ is a population-independent constant with $C_{2}\geq 0$ if $G_{\mu }$ is obtained from *all* single-genotype populations. The ruggedness metrics $\rho_{1}$ and $\rho_{2}$ therefore govern the rate at which the mean population fitness and its square relax towards the landscape average $\bar{f}$ and ${\bar{f}}^{2}$. In both cases, the decay rates can be interpreted as the ruggedness of the landscape by relating them to the different Fourier components from Fig 2: for large $\rho_{1}$ or $\rho_{2}$, higher-frequency terms dominate the corresponding decay, and for small $\rho_{1}$ or $\rho_{2}$, lower-frequency terms dominate. This observation is the phenomenon that our work exploits to estimate the landscape ruggedness through measurements of the mean fitness of a population as it accumulates mutations. Suppose that each population member is mutagenised with θ single-point mutations per generation on average, and the mean fitness is recorded as $F_{\mu_{1}}$, $F_{\mu_{2}}$, ..., $F_{\mu_{M}}$ with $\mu_{i}=\theta i$ over *M* generations. These values can be used to estimate the parameters *C*_1_, $\rho_{1}$, and $\bar{f}$ in Eq (2a). Equivalently, by squaring the mean-fitness trajectory for each starting population and then averaging the squared trajectories, they can be used to estimate *C*_2_, $\rho_{2}$, and ${\bar{f}}^{2}$ in Eq (2b). The analytical quantities $\rho_{1}$ and $\rho_{2}$ can be computed from Eq (1), whereas the fitted quantities are obtained by approximating finite observations with a single exponential decay. In the following, we denote decay rates fitted from finite, noisy observations by $\rho_{1}^{fit}$ for $F_{\mu }$ and $\rho_{2}^{fit}$ for $G_{\mu }$. We further distinguish between local estimates $\rho_{2}^{loc}$, obtained from one or a small number of starting genotypes, and global estimates $\rho_{2}^{glob}$, obtained after averaging over all, or a large representative set of, starting genotypes.

**Fig 3:**
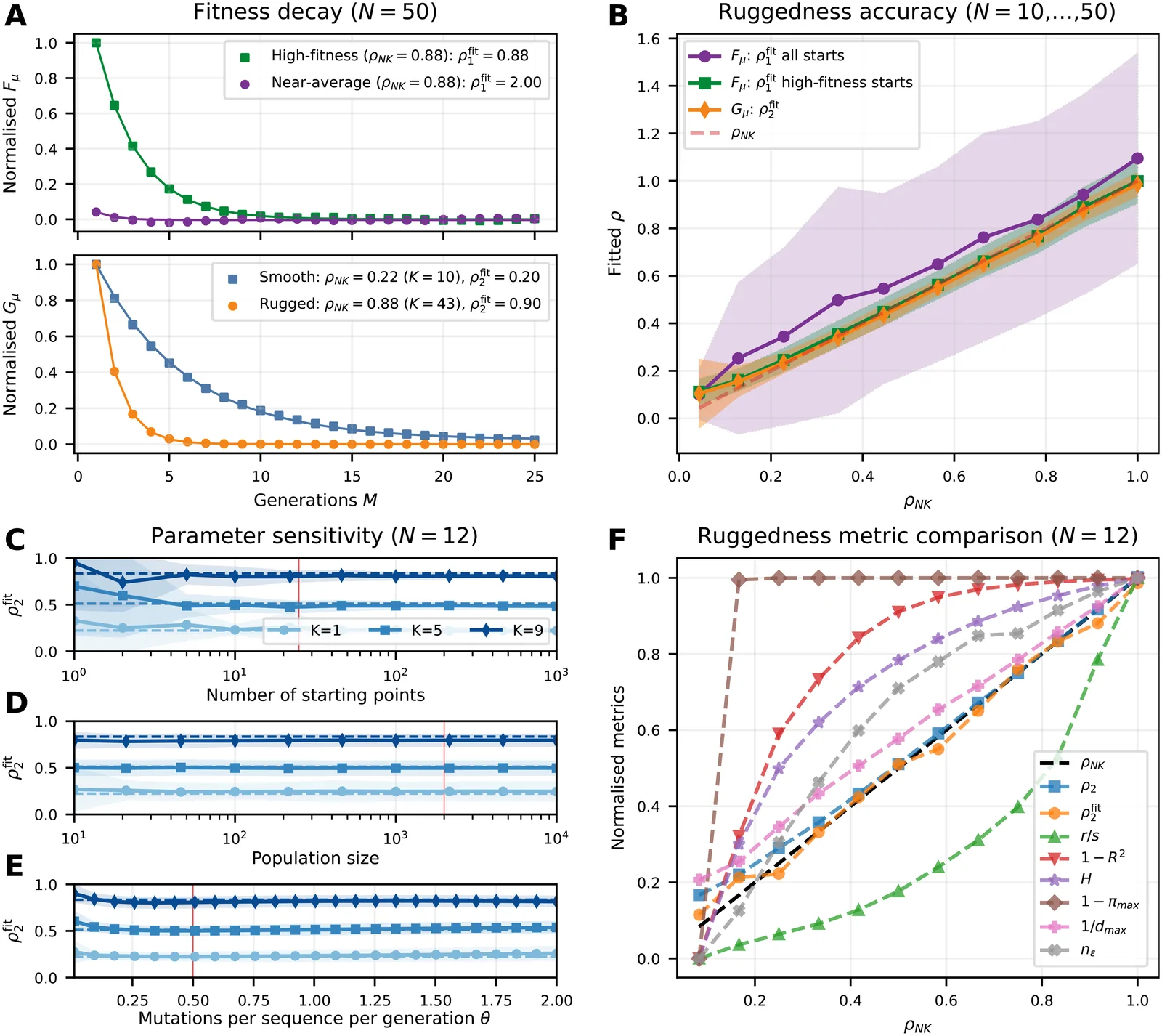
Fitness-decay estimates of landscape ruggedness on *NK* landscapes. **A:** Example decay trajectories on binary *NK* landscapes. The upper subpanel shows $F_{\mu }$ on a rugged landscape for one near-average-fitness start and one high-fitness start, selected from the starts closest to mean initial fitness and from the top 20% of initial fitness, respectively. The lower subpanel shows $G_{\mu }$ for a smoother landscape and the same rugged landscape; solid lines show exponential fits. **B:** Fitted decay rates across the *NK* grid (N=10,...,50; 100 *NK* pairs; 25 landscapes per pair; 25 starts per landscape). For $F_{\mu }$, $\rho_{1}^{fit}$ is computed either from all starts or from high-fitness starts. For $G_{\mu }$, $\rho_{2}^{fit}$ is computed from all starts. Points and bands show bin means and empirical standard deviations (SDs) across estimates in each $\rho_{NK}$ bin. **C–E:** Sensitivity of $\rho_{2}^{fit}$ for *N* = 12, K∈{1,5,9}, and 25 landscapes per *K*; red vertical lines denote the fixed parameter values used when varying the other parameters; shaded bands show empirical SDs across landscapes. **F:** Comparison of fitted decay rates and analytical ruggedness metrics on *N* = 12, binary *NK* landscapes with K=0,...,11. Analytical metrics are averaged over 50 landscapes per *K*; all ruggedness metrics are normalised for comparison. Unless otherwise stated, diffusion simulations used 25 starts per *NK* landscape, 5 replicate populations per start, mutation rate θ=0.5 (θ/N per site), and *M* = 25 generations. Replicate trajectories are averaged per start to obtain $F_{\mu }$; these per-start trajectories are squared and then averaged over starts to obtain $G_{\mu }$.

In homogeneous landscapes, such as *NK* landscapes, local and global notions of ruggedness broadly coincide. In particular, the epistatic interaction parameter *K* defines the closed-form ruggedness value $\rho_{NK}\;\begin{array}{c} \overset{\text{def}}{=}\;(K+1)/N \end{array}$, and the analytical decay rates satisfy $\rho_{1}\approx \rho_{2}\approx \rho_{NK}$ (see Eq (S2) in Section D of S1 Appendix). Fig 3A illustrates the behaviour of these two quantities on *NK* landscapes. For $F_{\mu }$, the exponential approximation is accurate when the starting population lies sufficiently far from the landscape average fitness, whereas populations starting close to the average exhibit little measurable decay and consequently less reliable fitted decay rates (top panel of Fig 3A). In general, $\rho_{1}$ represents a local notion of ruggedness associated with a particular starting population. By contrast, $G_{\mu }$ averages the squared fitness over starting populations, making the decay substantially more robust to the choice of starting genotype. In the bottom panel of Fig 3A, $\rho_{2}^{fit}$ is computed from 25 starting populations and matches $\rho_{NK}$. This behaviour is confirmed in Fig 3B, which shows decay rates computed on a grid of *NK* landscapes for N=10,...,50 and shows that $\rho_{2}^{fit}$ and $\rho_{1}^{fit}$ computed from high-fitness starting populations both correlate strongly with $\rho_{NK}$. Beyond *NK* landscapes, these metrics broadly coincide if the landscape is dominated by a single spectral frequency. In heterogeneous landscapes where more than one frequency dominates, however, local and global notions of ruggedness need not agree, and fitted estimates may differ from the corresponding analytical values.

Due to the noise associated with observing a randomly mutating population, the reliability of $\rho_{1}^{fit}$ and $\rho_{2}^{fit}$ estimates also depends on the parameters of the observed population, including the population size, the mutation rate θ, and for $G_{\mu }$, the number of averaged starting points. Fig 3C–3E analyses the sensitivity of $\rho_{2}^{fit}$ with respect to these parameters, where red vertical lines denote the fixed parameter values used when other parameters are varied, and dashed lines denote the analytical $\rho_{2}$. Fig 3C shows $\rho_{2}^{fit}$ estimates over an increasing number of starting points. If fewer than 10 starting points are used, $\rho_{2}^{fit}$ estimates can exhibit a large variance.

Fig 3D shows $\rho_{2}^{fit}$ estimates over increasing population sizes. For small population sizes (≤20), the ruggedness estimate exhibits higher variability due to noise in the mean population fitness. The effect of mutation rate is analysed in Fig 3E; for small θ, only a small fraction of the fitness decay curve in Fig 3A is captured, leading to an increased variability of $\rho_{2}^{fit}$. For very large θ (θ≫2), the mean population fitness decays more rapidly to the landscape average (comparable to the top panel of Fig 3A), again leading to a large variability of $\rho_{2}^{fit}$ since only a few datapoints characterise the decay. In practice, an estimate of the average mutation rate per generation is required, which is assumed to be constant across generations (see Eq (3) in Methods). If the mutation rate per generation θ is not exactly known but estimated as $\tilde{\theta }$, $\rho^{fit}$ will scale with $\theta /\tilde{\theta }$ as long as the mutation rate is constant across generations, e.g., if the actual θ is twice as high as $\tilde{\theta }$ then $\rho^{fit}$ will be twice as large. As such, if the mutation rate error is constant across analysed landscapes, $\rho_{1}^{fit}$ and $\rho_{2}^{fit}$ values remain comparable, i.e., the ordering resulting from the metric is preserved under an estimation error.

To further validate the decay rates from Eq (1), Fig 3F provides a comparison between $\rho_{2}$ and other ruggedness measures across *NK* landscapes (see Section C of S1 Appendix). The other ruggedness measures are the roughness to slope ratio *r*/*s* [4], the landscape *R*^2^ measure [8], the spectral entropy *H*, number of paths to the global maximum from the opposite position on the landscape $\pi_{max}$ [7], the distance to the nearest local maximum $d_{max}$ [6], and the local epistasis metric $n_{\epsilon }$ [8]. Fig 3F shows that although all metrics increase with $\rho_{NK}$, they express either concave (*r*/*s*), linear (decay rates and $1/d_{max}$), or convex curves (all other metrics). Because all metrics increase with *K*, this allows one to compare the ordering (w.r.t. ruggedness) of landscapes across metrics, but the differences in curve shapes prevent comparison of distances (i.e., quantitative ruggedness differences) between landscapes across metrics. However, one can mathematically prove that the decay rates directly correlate with $\rho_{NK}$ (see Eq (S2) in Section D of S1 Appendix), and the same relationship holds regardless of *N*. Overall we believe this provides a second benefit of our methodology—that it provides an interpretable measure of ruggedness that has immediate meaning in the widely studied *NK* landscapes.

### Ruggedness inference on empirical landscapes

**Fig 4:**
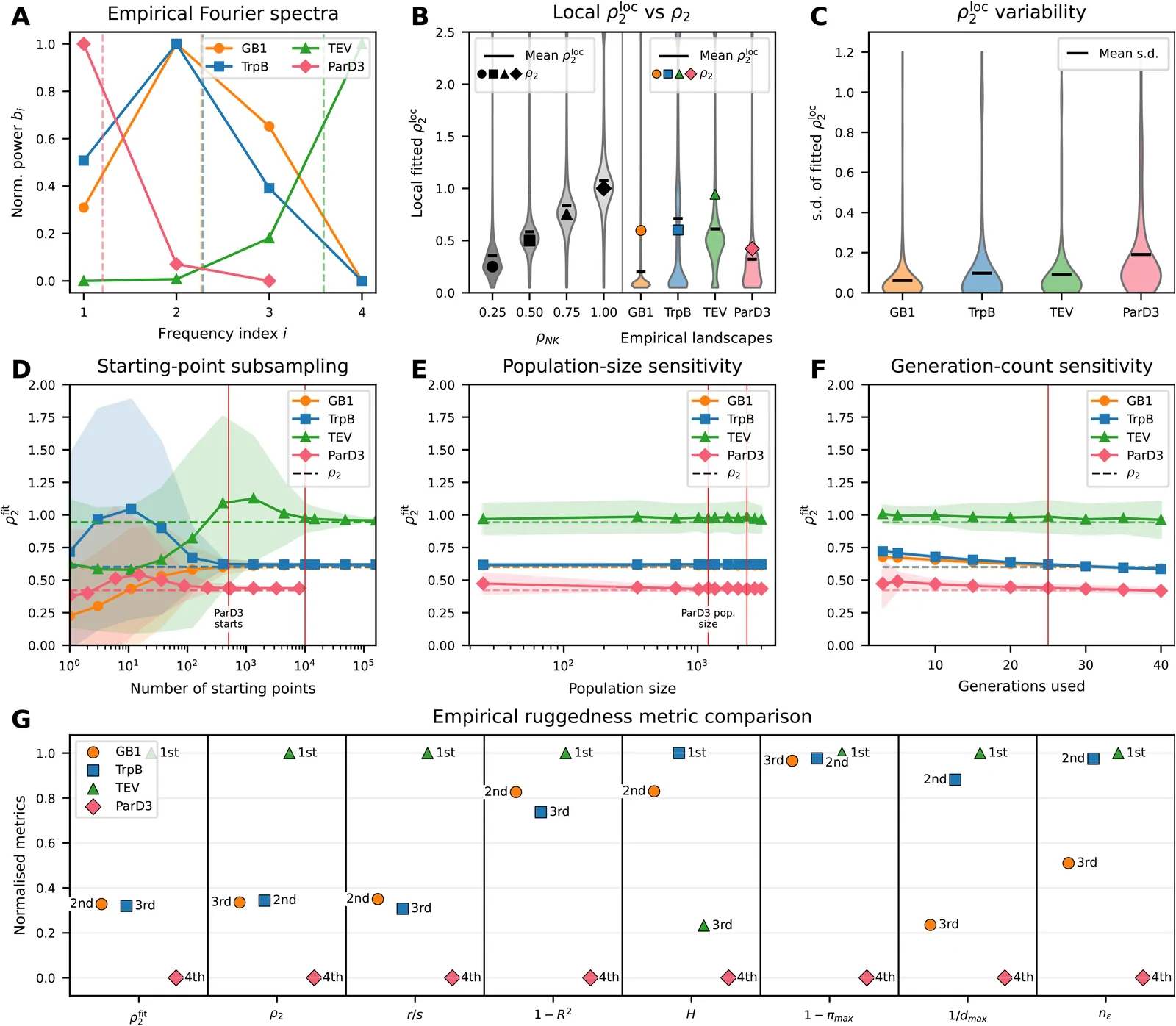
Ruggedness estimation on empirical landscapes. **A:** Fourier spectra of the complete GB1, TrpB, TEV and ParD3 landscapes computed by GFT; dashed lines indicate the frequency centroid $\rho_{2}\times d/A$. **B:** Violin plots showing $\rho_{2}^{loc}$ obtained by fitting the fitness decay from each individual starting genotype. Markers show the analytical $\rho_{2}$. **C:** Violin plots showing replicate variability in $\rho_{2}^{loc}$: for each landscape, estimates were repeatedly computed from bootstrap samples of 10 starts and 5 replicates per start (250 bootstrap samples). **D–F:** Sensitivity of $\rho_{2}^{fit}$; red vertical lines denote the fixed parameter values used when varying the other parameters; dashed horizontal lines denote $\rho_{2}$; shaded bands show SDs across bootstrap samples. **D:** Starting-point subsampling. Estimates were computed from increasing numbers of starting genotypes using 1000 bootstrap samples. **E:** Population-size sensitivity using 250 bootstrap samples per population size. **F:** Generation-count sensitivity using 250 bootstrap samples. **G:** Comparison of decay rates and other ruggedness metrics applied to the empirical landscapes; metrics are normalised for comparison. Unless otherwise stated, diffusion simulations used mutation rate θ=0.1 per sequence (θ/N per site), 10 replicate populations per start and 40 simulated generations. Global fits use the first 25 generations and, unless varied, 10,000 sampled starts for GB1, TrpB and TEV or 500 starts for ParD3.

The performance of SLIDE on empirical landscapes was assessed *in silico* by testing it on four experimentally measured landscapes: GB1 (4 sites) [2], ParD3 (3 sites) [24], TrpB (4 sites) [25] and TEV (4 sites) [26]. The Fourier spectra of each landscape are shown in Fig 4A, with the dashed vertical lines denoting the frequency centroids computed from the analytical $\rho_{2}$ as $\rho_{2}\times d/A$. Here, in contrast to *NK* landscapes, the heterogeneity of empirical landscapes or—in other words—the spread of spectra makes it necessary to distinguish between $\rho_{2}^{fit}$ estimated from one or a few starting points (denoted by $\rho_{2}^{loc}$) and $\rho_{2}^{fit}$ estimated from all or a large set of starts (denoted by $\rho_{2}^{glob}$). Fig 4B compares $\rho_{2}^{loc}$ obtained from single-genotype starting populations (shaded) with the analytical $\rho_{2}$ obtained from Eq (1b). On *NK* landscapes, these values broadly correspond, indicating that *NK* landscapes are effectively homogeneous. On empirical landscapes, $\rho_{2}^{loc}$ and $\rho_{2}$ do not correspond. However, the starting population-dependent estimates may still be used to measure the ruggedness of an initial population ν. This is shown in Fig 4C, which shows the SD of $\rho_{2}^{loc}$ estimates over 5 replicates with the fitness decay computed from a population comprising 10 starting points. Variability between estimates from the same starting populations is much smaller than in Fig 4B.

Practically, in order to obtain a global property from empirical landscapes despite their increased heterogeneity, one must sample multiple starting points. Despite the added labour required to measure multiple starting points, such a configuration remains scalable as it requires random mutagenesis only, something which is commonly conducted in a pooled format for library generation (e.g., using error-prone PCR). Fig 4D shows how the fitted decay rate $\rho_{2}^{fit}$ varies as the number of starting points is increased. As it approaches the total number of starting points, the decay rate converges from $\rho_{2}^{loc}$ (which may vary depending on starting populations) towards $\rho_{2}^{glob}$. The difference between $\rho_{2}^{glob}$ and $\rho_{2}$ (dotted) depends on the “spread” of the spectrum; if the power is concentrated in a single frequency then the fitted global value coincides with Eq (1b). Generally, $\rho_{2}^{glob}$ and $\rho_{2}$ are related via a first-order approximation of $G_{\mu }$ (see Methods). Fig 4E shows how $\rho_{2}^{fit}$ varies with population size, and Fig 4F with number of generations sampled to obtain the fitness decay. In Fig 4F, the mutation rate is θ=0.1 per sequence, so that *M* generations correspond to μ=θM accumulated mutations. The principal determinant of whether an estimate approaches $\rho_{2}^{glob}$ is the number of starting points sampled.

Fig 4G compares other ruggedness measures (described in Section C of S1 Appendix) across the empirical landscapes. Local epistasis ($n_{\epsilon }$), distance to local maximum ($d_{max}$), and paths to global maximum ($\pi_{max}$) agree with the analytical metric $\rho_{2}$ in terms of their ordering of all the empirical landscapes tested. The landscape *R*^2^ and roughness to slope ratio (*r*/*s*) agree on the two extremes (TEV as the most rugged, ParD3 as the least), but place GB1 and TrpB in a different order. Similarly, $\rho_{2}$ groups together TrpB and GB1 landscapes as having a similar, intermediate ruggedness, whereas other methods separate these landscapes, or locate them very close to the TEV landscape. The general agreement between other ruggedness metrics and the ordering produced by $\rho_{2}$ on empirical landscapes provides increased confidence in the metric, which has the major advantage over all others of being experimentally measurable without detailed sequencing data (see Section E of S1 Appendix for a biological interpretation of these ruggedness values).

The analyses in Figs 3 and 4 assume uniform, unbiased mutation and are conducted directly on amino-acid landscapes, with *A* = 20 for the empirical landscapes. To assess the robustness of the approach under more realistic conditions, we further analysed ruggedness inference under biased mutation and controlled landscape perturbations, as described in the Methods and shown in Fig 6. The decay-based framework extends to weighted-undirected and directed mutation models, provided that the mutation process converges to a stationary distribution. Across the mutation models considered, the corresponding ruggedness estimates changed only modestly, indicating that the inferred ruggedness was largely robust to the considered mutation spectra. We additionally evaluated $\rho_{2}$ after introducing minimum-fitness regions, neutral ridges, and modular structure into *NK* landscapes. The metric responded systematically to each perturbation; for example, increasing the fraction of minimum-fitness genotypes increased the inferred ruggedness. However, because such perturbations generally introduce spatial heterogeneity, estimating a global ruggedness value may require fitness-decay measurements from a larger number of starting points.

### Optimising DE outcomes with SLIDE

Inferring the ruggedness of a fitness landscape prior to DE allows informed decisions to be made about the DE strategy [2,3]. Here, as an illustrative application of SLIDE, we interface the decay-rate-based ruggedness estimate with the strategy framework developed in our previous work [10]. In that framework, combinations of two parameters—a fitness-independent selection probability (base chance) and population splitting—were evaluated on *NK* landscapes to determine the optimal strategy as a function of landscape ruggedness. We compare the resulting ruggedness-adapted strategy with two fixed-parameter strategies that can be represented within the same parameter space. The baseline strategy uses no population splitting and selects the top 20% of candidates. The high-exploration (HE) strategy uses high population splitting and a base chance of 20%, producing selection behaviour that approaches an almost random policy. These fixed strategies therefore represent the two extremes of exploitation and exploration, providing benchmarks for assessing the benefit of adapting this balance to the inferred ruggedness.

**Fig 5:**
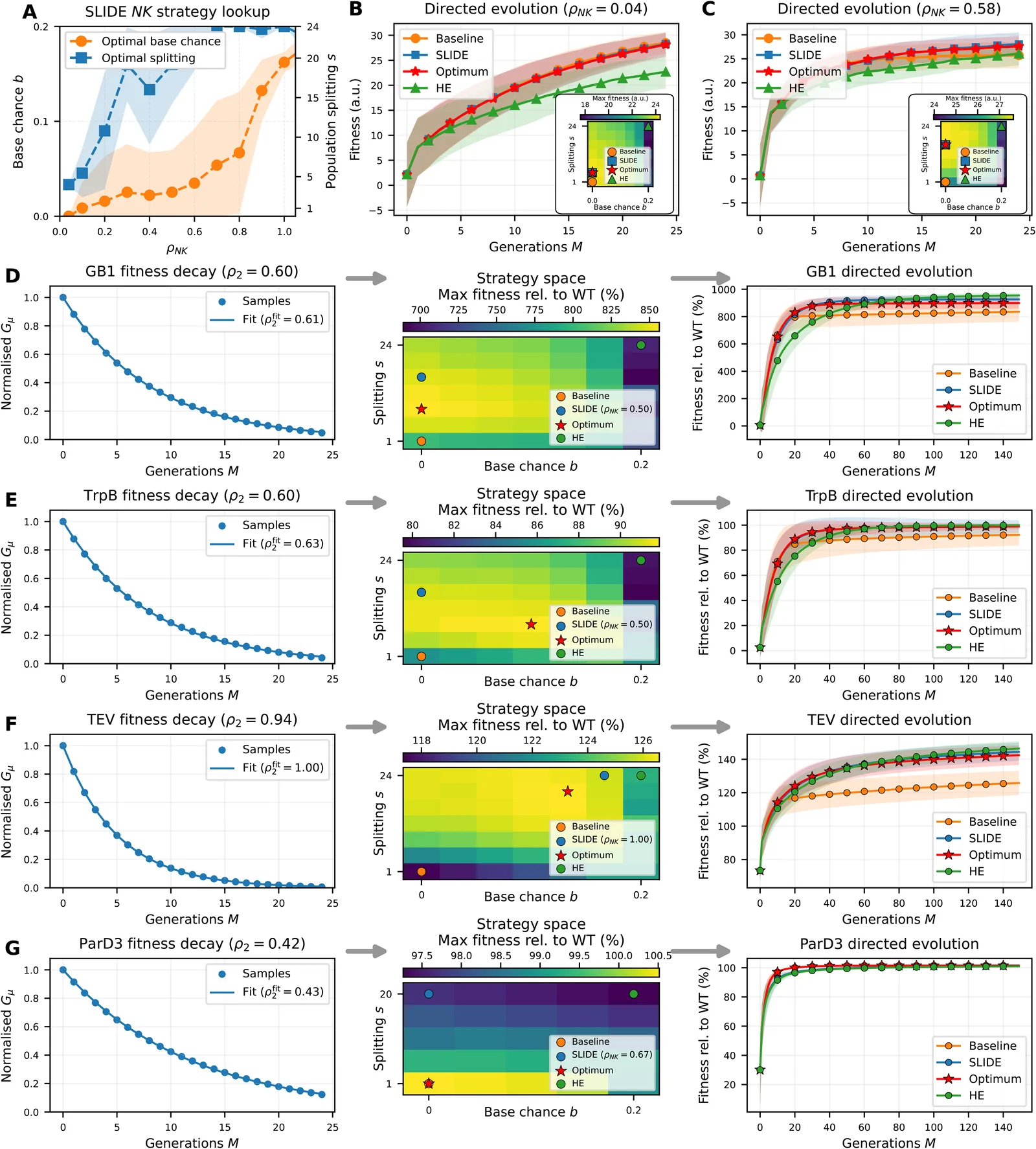
DE using SLIDE on *NK* and empirical landscapes. **A:** Representation of the SLIDE look-up table for *NK*. Generated from 100 (*N*,*K*) combinations with 10≤N≤50. For each combination, all strategies were evaluated for *M* = 25 generations using 200 independently generated landscapes and 25 replicates per strategy. Base chance options = {0.00,0.03,0.06,0.10,0.13,0.16,0.19}, splitting options = {24,20,...,4,1}. Lines show the mean optimal parameter values among (*N*,*K*) combinations grouped by $\rho_{NK}$; shaded regions show one SD across those combinations. **B–C:** SLIDE applied to smooth (*N* = 45,*K* = 1) and rugged (*N* = 45,*K* = 25) *NK* landscapes. Lines show mean fitness over 100 simulations on one landscape; shading indicates ±1 SD. Strategy spaces show mean fitness after 25 generations across 100 random starts and 300 replicates per start, with markers indicating the strategies selected by each approach. Population size = 1200; mutation rate θ=0.1/N per site per generation. **D–G:** SLIDE applied to the GB1, TrpB, TEV and ParD3 empirical landscapes. Decay curves were estimated from 10,000 starting genotypes and 10 replicates (θ=0.1/N per site; population size = 2500). Strategy spaces show mean fitness after 25 generations across 100 random starts and 300 replicates per start, computed on the corresponding empirical landscape; DE trajectory lines show the corresponding means over 150 generations, with shading indicating ±1 SD across starts. SLIDE strategies were selected using *A* = 20 *NK* look-up tables with *N* = 4. For **G**, *N* = 3, 8,000 decay starts, population size = 60, and splitting options {20,15,10,5,1} were used. HE, high exploration.

To explore the relationship between landscape ruggedness and optimal strategy, we evaluated 100 distinct (*N*,*K*) combinations at fixed population size and mutation rate (Sections A and B of S1 Appendix). For each combination, all 49 strategy combinations were evaluated for *M* = 25 DE generations on 200 independently generated *NK* landscapes, with 25 simulation replicates per strategy (10≤N≤50). Fig 5A shows the mean optimal base-chance and splitting values as a function of $\rho_{NK}$, with shaded regions indicating one SD across (*N*,*K*) combinations grouped by $\rho_{NK}$. Note that the optimal strategy is also dependent on the number of generations used in DE—more generations favour more explorative strategies as shown in S4 Fig. Ideally, the SLIDE look-up table should therefore be generated *in silico* using the anticipated number of generations, mutation rate, and population size of the experiment.

Because $\rho_{1}\approx \rho_{2}\approx \rho_{NK}$ on *NK* landscapes, these results provide a SLIDE look-up table indexed by ruggedness. A fitted estimate, $\rho_{1}^{fit}$ or $\rho_{2}^{fit}$, can therefore be used to select an appropriate combination of DE parameters. Two examples of DE trajectories on *NK* landscapes are displayed in Fig 5B–5C. These figures additionally show how close each selected strategy is to the optimal strategy, the baseline strategy, and the HE strategy. Fig 5B displays a smooth landscape ($\rho_{NK}=0.04$), where the strategy selected by SLIDE is highly similar to the baseline strategy (which is already near the optimum). Fig 5C, however, displays a more rugged landscape ($\rho_{NK}=0.58$), on which the baseline strategy is highly sub-optimal and gets trapped at a local optimum within 10 generations. SLIDE, on the other hand, selects the optimal strategy within the strategy space and achieves >15 % higher fitness than the baseline strategy after 50 generations. The corresponding results in S1 Fig demonstrate the robustness of this trend across alternative landscape models other than *NK* (Rough Mount Fuji and a stochastic block model).

A similar approach was then applied to the four combinatorially complete empirical landscapes GB1, TrpB, TEV and ParD3 in Fig 5D–5G. The left-hand panels show the decay rate estimation from $G_{\mu }$ using 10,000 starting points. The middle panels evaluate all possible strategies on the corresponding empirical landscape and show the strategy selected via SLIDE; the SLIDE look-up table, as displayed in Fig 5A, was re-generated using *NK* landscapes of the same dimensions as each empirical landscape (*A* = 20, *N* = 4 or *N* = 3 for ParD3) and for *M* = 25 DE generations. SLIDE outperformed the baseline strategy in all examples, apart from ParD3, for which the baseline strategy was optimal (and therefore SLIDE selected the same parameters). After 150 generations on GB1, TrpB and TEV, it achieved relative fitness gains of 24.9% (*p* < 0.001), 22.6% (*p* < 0.01), and 44.2% (*p* < 0.001), respectively, over the baseline approach, which commonly gets trapped at local maxima. These improvements suggest that adapting the DE strategy to the inferred landscape structure can substantially improve optimisation performance. Consequently, integrating the ruggedness estimation framework proposed here with existing DE workflows may yield similar gains compared with fixed-parameter strategies.

## Discussion

In this paper, we showed that in the absence of selection pressure, the average fitness decay of a population accumulating mutations can be approximated by an exponential curve whose fitted decay rate $\rho_{1}^{fit}$ approximates the analytical, genotype-dependent metric $\rho_{1}$. The analogous squared-fitness decay rate $\rho_{2}^{fit}$ approximates the genotype-independent metric $\rho_{2}$, which is linked to the normalised Dirichlet energy of the graph Laplacian and quantifies squared fitness variation across mutation neighbours. Under unbiased mutations, the decay rate $\rho_{2}$ reduces to the Fourier power-weighted average frequency of the Hamming graph Laplacian. Under biased mutation spectra, the same decay-based construction yields a metric associated with the effective mutation operator rather than the unweighted Hamming graph. Thus, if the mutational or experimental protocol induces non-uniform or selection-coupled dynamics—for example through codon-level mutation biases, viability filtering, repair biases, or growth-coupled sampling—the fitted decay rate should be interpreted as a protocol-dependent dynamical descriptor of the effective landscape explored by the population. For DE control, this effective ruggedness remains relevant, since it characterises the search process experienced under the protocol being used.

We validated the decay rate estimation *in silico* on synthetic *NK* landscapes and empirical datasets. Due to their homogeneity, the estimation procedure on *NK* landscapes is robust to the choice of mutation rate, population size, decay curve length, and number of starting genotypes, and the analytical, fitted, local, and global decay rates broadly correspond. The primary requirements are that the starting genotype is sufficiently far from the landscape average, and that the mutation rate is sufficiently large to induce observable decay, yet not so large that observed fitnesses lose temporal correlation.

We further tested SLIDE on systematically perturbed landscapes containing minimum-fitness regions, neutral ridges, and modular structure. As expected, these perturbations alter the Fourier spectrum and are reflected in the inferred ruggedness: minimum-fitness regions introduce sharp discontinuities and shift spectral power towards higher frequencies, whereas neutral ridges and modularity suppress higher-order variation and shift power towards lower frequencies. These results demonstrate that the inferred ruggedness captures the spectral consequences of diverse structural landscape features. However, because the metric provides a scalar summary of the spectrum, it does not by itself identify which particular landscape feature produced a given ruggedness value.

On more heterogeneous empirical landscapes, the ruggedness estimation requires more starting points to obtain a global estimate. However, representing the complete nucleotide genotype space through the nucleotide-to-amino-acid mapping spreads the landscape spectra over a broader range of frequencies, so that fewer starting genotypes are required to obtain a stable, global estimate at nucleotide level than at amino-acid level. Our simulations showed that for the considered empirical landscapes, estimating ruggedness at the amino-acid level required approximately 500–10,000 starting genotypes (0.25% to 6.25% of the landscape), whereas estimation at the nucleotide level required only 100 starting genotypes ($<6\times 10^{-4}\;\%$ of the landscape). At the same time, however, the resulting ruggedness values are more closely spaced, so greater estimation accuracy is required to distinguish reliably between landscapes. By contrast, only 3–5 replicates were required to estimate a local ruggedness metric $\rho_{1}^{fit}$ or $\rho_{2}^{loc}$ accurately. Local metrics have not been analysed further here, but could be combined with a downstream DE selection strategy or used to measure the genetic stability of an individual genotype.

Using the decay rate as a ruggedness metric, we introduced SLIDE (Sequence-free Landscape Inference for Directed Evolution), a framework that estimates landscape ruggedness from average fitness decay curves. Conceptually, SLIDE aligns with broader work employing graph-based methods to extract structural properties of fitness landscapes [3,27–29]. Unlike existing methods, SLIDE requires only large-scale phenotypic data alongside a mutation rate estimation—removing the need for large-scale sequence-level information. As long as fitness can be coupled to a measurable phenotype such as fluorescence, SLIDE enables ruggedness estimation from bulk population-level measurements and knowledge of the average mutation rate. This feature makes SLIDE scalable and experimentally accessible.

By using SLIDE’s ruggedness estimate to tune DE parameters, we demonstrated up to a 44.2% improvement in DE performance compared to the top-proportion selection baseline, even when $\rho_{2}^{fit}$ was only within ±25 % of $\rho_{2}^{glob}$. These gains arise because the inferred ruggedness enables the directed evolution strategy to be adapted to the underlying landscape structure, rather than relying on fixed mutation and selection parameters. While this paper applied DE strategies from [10], the ruggedness metric is broadly compatible with a wide range of advanced DE approaches, including AI-driven workflows. For example, in active learning-assisted DE [14], machine learning techniques are used to guide the selection, balancing exploration of unknown phenotypes with exploitation of high-performing variants. Here, the ruggedness metric obtained from SLIDE could be used to modulate the balance of exploration and exploitation. Similarly, in cluster learning-assisted DE [30], SLIDE could be used to tailor clustering granularity.

Although this work focused primarily on the theoretical and computational aspects of SLIDE, the framework is designed to be experimentally implementable. Local ruggedness estimation requires only a small number of replicate trajectories, whereas global estimation remains substantially more demanding because it must average over heterogeneity across the landscape. However, the number of required starting genotypes is significantly reduced at the nucleotide level, and even global SLIDE estimation requires only a fraction of the measurements needed to construct and sequence a combinatorially complete empirical landscape. The key requirement is a biological protocol for introducing mutations without applying selection pressure and with a mutation rate that is approximately constant across generations on average. One example is random *in vitro* mutagenesis, which allows targeted exploration of specific regions of the genotype space. Mutant libraries generated in this way can be expressed in cells, with fitness linked to a measurable phenotype such as fluorescence. By iteratively measuring average fluorescence across generations and estimating mutation accumulation, or by simultaneously producing samples with increasing levels of mutation, it becomes possible to construct fitness-decay curves and apply SLIDE in a laboratory setting. Such an assay could be conducted in several 96-well plates, or at larger scales using automated liquid-handling systems, and could also be implemented in a pooled format using mother-machine microfluidics. Mother machines are microfluidic chips capable of long-term culture of up to 10^6^ individual cells in spatially segregated chambers that eliminate growth-based competition [31,32]. In such a format, one need only introduce the original variant library into the device and observe the cells while inducing mutagenesis, using chemical mutagens, UV [33] or even targeted *in vivo* mutagenesis methods [34].

Several limitations remain. First, the present framework primarily considers fitness measurements that are decoupled from host or variant growth. This requirement could be relaxed by incorporating selection directly into the population dynamics. In general, selection can be represented as an additional operator acting on genotype frequencies—for example, through genotype-dependent growth or explicit enrichment rules—and combined with the mutation operator to yield mutation–selection dynamics. The observed fitness trajectories would then be governed by these combined dynamics, such that SLIDE estimates a ruggedness metric associated with the mutation–selection process rather than with a freely diffusing population. One approximation is to absorb selection into an effective biased mutation spectrum, as described in Methods. When selection induces only weak bias, the resulting metric remains close to the unbiased one; more generally, it quantifies ruggedness along the biased dynamics through which the landscape is actually traversed. This could allow the decay rate to be identified online and used as a feedback signal, either to update the ruggedness estimate or to adapt DE strategy parameters directly. For instance, under truncation selection, the early fitness gradient could be linked computationally and analytically to landscape ruggedness and structure (cf. Fig 5). Such extensions could enable SLIDE to operate *during* DE in settings where mutation and selection act simultaneously, including continuous *in vivo* mutagenesis [34] in small-scale bioreactors.

Second, future work should consider more general mutation operators. In particular, some mutation graphs may not admit a unique stationary distribution, for example when they are reducible or contain sink nodes. In such cases, the long-term fitness average and the inferred decay behaviour may depend on the initial population, and the present interpretation of the ruggedness metric would need to be extended accordingly.

Third, we analysed biased mutation, selection-related effects, and structural landscape perturbations largely in isolation. We did not systematically study their combined effects, for example when biased mutation or selection acts on landscapes containing neutral ridges. These interactions could alter both the effective spectrum and the spatial heterogeneity of the decay process, and therefore the relationship between local and global ruggedness estimates. A unified treatment of mutation, selection, and landscape structure is beyond the scope of the present work but represents an important direction for future study.

Finally, although the empirical landscapes provide combinatorially complete genotype–fitness maps, they cover only three- to four-site protein subspaces, and all validation presented here is computational. The present results therefore provide limited evidence for performance in larger, sparsely sampled, and structurally constrained protein spaces or in experimental DE campaigns. Addressing this limitation will require both experimental validation and theoretical landscape models that can be tuned to reproduce a broader range of real-world properties. Developing such models, potentially using generative approaches or other data-driven methods, represents an important direction for future research.

In summary, SLIDE offers a novel method for estimating the ruggedness of protein fitness landscapes using average phenotypic data alongside mutation rate estimation. These ruggedness estimates can inform experimental strategies in DE, helping to tailor protocols to the underlying structure of the landscape. Next to DE, SLIDE’s ruggedness metric could be extended to characterise more complex landscapes, such as those underlying synthetic biological circuits. Large-scale mapping of ruggedness in such systems could provide a quantitative basis for designing circuits with greater genetic stability and functional robustness, complementing recent efforts to understand and engineer the evolutionary potential of synthetic constructs [35].

## Methods

### Decay rate metric

To show why the fitness decay rates $\rho_{1}$ and $\rho_{2}$ in Eq (2a) can be used as ruggedness metrics, we give a brief overview of spectral landscape theory. We will represent a population accumulating single-point mutations as a stochastic signal in a graph, modelling mutagenesis and genotype space. For a gene of length *N*, we denote the genotype space by $𝒢\;\begin{array}{c} \overset{\text{def}}{=}\;{\mathbb{Z}}_{A}^{N} \end{array}$ (the space of positive integers up to *A*, in a vector of *N* entries), containing $|𝒢|=A^{N}$ genotypes $x_{1},\;...,x_{A^{N}}$. Each genotype can be written as $x_{i}=(\sigma_{1}^{i},\;...,\sigma_{N}^{i}{)}^{\text{T}}$, where $\sigma_{j}^{i}\in {\mathbb{Z}}_{A}$ is the allele at locus *j*. For example, if *A* = 20 represents the number of possible amino acids at each position in a protein of length *N*, the landscape consists of $A^{N}$ distinct genotypes. These genotypes can be represented as nodes in a graph, with each node being assigned a fitness value $f_{i}\;\begin{array}{c} \overset{\text{def}}{=}\;f(x_{i}) \end{array}$, where f:𝒢↦ℝ characterises the fitness landscape. A population is represented by $\nu =(\nu_{1},...,\nu_{A^{N}}{)}^{\text{T}}\in {\mathbb{R}}^{A^{N}}$, where each $\nu_{i}$ gives the frequency of genotype $x_{i}$. Equivalently, ν can be defined as a probability distribution, in which case $\sum_{i}\nu_{i}=1$ with all $\nu_{i}\in [0,1]$. The mean population fitness is then computed as $F=f^{\text{T}}\nu =\sum_{i}f_{i}\nu_{i}$, where $f=(f_{1},...,f_{A^{N}}{)}^{\text{T}}\in {\mathbb{R}}^{A^{N}}$ is the stacked vector of fitness values.

The edges of the graph connect genotypes that differ by a single mutation, so that every node is connected to $d\;\begin{array}{c} \overset{\text{def}}{=}\;N(A-1) \end{array}$ other nodes; such a graph is also referred to as a Hamming graph [36]. For node transitions, we define the single-mutation operator 𝒟 to map an initial population ν to 𝒟(ν), where every population member has undergone a single, random and equally likely mutation (see Section A of S1 Appendix for details on our mutation model). It can be shown that 𝒟(ν)=Dν, where $D=D^{\text{T}}\in {\mathbb{R}}^{A^{N}\times A^{N}}$ is a row-stochastic matrix obtained from multiplying the adjacency matrix of the graph with 1/*d*.

We assume that the number of mutations that occurs in one mutagenesis round is Poisson distributed with mean θ, so that the number of mutations $X_{M}$ occurring between *M* rounds is Poisson distributed with mean $M\theta \;\begin{array}{c} \overset{\text{def}}{=}\;\mu \end{array}$. The population is therefore expected to evolve as


$$\begin{array}{c} {𝔼}_{X_{M}}\left[{𝒟}^{X_{M}}(\nu )\right]=\left(\sum_{k=0}^{\infty }Pr(X_{M}=k)D^{k}\right)\nu =\left(\sum_{k=0}^{\infty }e^{-M\theta }\frac{(M\theta {)}^{k}}{k!}D^{k}\right)\nu =\left(\sum_{k=0}^{\infty }e^{-\mu }\frac{\mu^{k}}{k!}D^{k}\right)\nu \;\begin{array}{c} \overset{\text{def}}{=}\;{ℳ}^{M}\nu . \end{array} \end{array}$$ (3)

Our mutation operator ${ℳ}^{M}$ can be understood as the transition function of a continuous-time Markov chain with a single genotype randomly mutating at rate θ. It can be simplified using matrix exponentials as


$$\begin{array}{c} {ℳ}^{M}=\sum_{k=0}^{\infty }e^{-\mu }\frac{\mu^{k}}{k!}D^{k}=e^{-\mu }\sum_{k=0}^{\infty }\frac{(\mu D{)}^{k}}{k!}=e^{-\mu }e^{\mu D}=e^{-\mu (I-D)}\;\begin{array}{c} \overset{\text{def}}{=}\;e^{-\frac{\mu }{d}\Delta }, \end{array} \end{array}$$ (4)

where we substituted the graph Laplacian $\Delta =\Delta^{\text{T}}\;\begin{array}{c} \overset{\text{def}}{=}\;d(I-D) \end{array}$. The mean fitness $F_{\mu }$ of our population after an average of μ=Mθ mutations is therefore


$$\begin{array}{c} F_{\mu }=f^{\text{T}}\left({ℳ}^{M}\nu \right)=f^{\text{T}}e^{-\frac{\mu }{d}\Delta }\nu . \end{array}$$ (5)

If necessary, the dependency on the initial population ν can be omitted by taking the expectation of Eq (5) over many random starting populations. In particular, if ν is sampled uniformly from single-genotype populations, so that $𝔼[\nu \nu^{\text{T}}]=I/A^{N}$, we can compute ${𝔼}_{\nu }[F_{\mu }^{2}]$ as:


$$\begin{array}{c} G_{\mu }\;\begin{array}{c} \overset{\text{def}}{=}\;{𝔼}_{\nu }[F_{\mu }^{2}]=f^{\text{T}}e^{-\frac{\mu }{d}\Delta }{𝔼}_{\nu }[\nu \nu^{\text{T}}]e^{-\frac{\mu }{d}\Delta^{\text{T}}}f=\frac{1}{A^{N}}f^{\text{T}}e^{-\frac{2\mu }{d}\Delta }f. \end{array} \end{array}$$ (6)

If ν is sampled from a smaller subset with $𝔼[\nu \nu^{\text{T}}]=\Sigma_{\nu }$, $G_{\mu }=f^{\text{T}}e^{-\frac{\mu }{d}\Delta }\Sigma_{\nu }e^{-\frac{\mu }{d}\Delta^{\text{T}}}f$. Eqs (5) and (6) can be further simplified by considering the special structure of a Hamming graph. Its real-valued, symmetric Laplacian admits a unitary Fourier basis as an eigenbasis [37], so that Δ can be factorised as $\Delta =P\Lambda P^{*}$, where $P\in {\mathbb{C}}^{A^{N}\times A^{N}}$ satisfying $P^{*}P=I$ contains the eigenvectors, and $\Lambda =\text{diag}(\lambda_{1},...,\lambda_{A^{N}})\in {\mathbb{R}}^{A^{N}\times A^{N}}$, with the eigenvalues ordered as $\lambda_{1}\leq \lambda_{2}\leq ...\leq \lambda_{A^{N}}$. Analogous to the standard Fourier transform, the eigenvalues capture a frequency, and the columns of *P* are eigenfunctions, each of which can be interpreted as eigenlandscapes of increasing ruggedness for increasing $\lambda_{i}$. For the Hamming graph Laplacian, there are *N* + 1 unique eigenvalues with value $\lambda_{i}=A\times i$ and multiplicity (Ni)(A−1)i, i=0,1,...,N [38]. The matrix *P* may be chosen as $P_{j,i}\;\begin{array}{c} \overset{\text{def}}{=}\;\chi_{j}(x_{i})/\sqrt{A^{N}} \end{array}$, where $\chi_{j}(x_{i})\;\begin{array}{c} \overset{\text{def}}{=}\;\exp(2\pi i\langle r_{j},x_{i}\rangle /A) \end{array}$ is the Fourier basis function, $r_{j}\in 𝒢$ the frequency index, and $\langle r_{j},x_{i}\rangle$ the dot product defined on 𝒢 (dot product modulo *A*) [37]. For $\lambda_{0}=0$, the eigenlandscape is a constant equal to the landscape average $\bar{f}\;\begin{array}{c} \overset{\text{def}}{=}\;1^{\text{T}}f/A^{N} \end{array}$, where $1\;\begin{array}{c} \overset{\text{def}}{=}\;(1,...,1{)}^{\text{T}} \end{array}$. For $\lambda_{1}=A$, the landscape is linear, i.e., depends only on the allele $\sigma_{j}$ at a single locus *j* (Fig 2C). For larger eigenvalues, the eigenlandscapes are rugged, i.e., they depend on the allele at multiple loci (exactly *i* different loci for $\lambda_{i}=Ai$). From these eigenvalue-eigenvector pairs, it also follows that $\lim_{\mu \to \infty }F_{\mu }=\bar{f}$ and $\lim_{\mu \to \infty }G_{\mu }=f^{\text{T}}11^{\text{T}}f/(A^{N}{)}^{2}={\bar{f}}^{2}$. In other words, if we sample uniformly from all single-genotype populations and average the fitness trajectories via ${𝔼}_{\nu }[F_{\mu }^{2}]$, the resulting $G_{\mu }$ decays from $G_{0}=f^{\text{T}}f/A^{N}$ exponentially to ${\bar{f}}^{2}$ as μ→∞.

The special eigenbasis of the Hamming graph allows the mean fitness decay to be broken down into contributions of different frequencies. Substituting $\Delta =P\Lambda P^{*}$ in Eqs (5) and (6) and defining $\hat{f}\;\begin{array}{c} \overset{\text{def}}{=}\;P^{*}f \end{array}$ and $\hat{\nu }\;\begin{array}{c} \overset{\text{def}}{=}\;P^{*}\nu \end{array}$ yields:


$$\begin{array}{cc} F_{\mu } & ={\hat{f}}^{*}e^{-\frac{\mu }{d}\Lambda }\hat{\nu }=\sum_{i=0}^{N}e^{-\frac{\mu \lambda_{i}}{d}}(\sum_{j\in {ℐ}_{i}}{\hat{f}}_{j}^{*}{\hat{\nu }}_{j})\;\begin{array}{c} \overset{\text{def}}{=}\;\sum_{i=0}^{N}e^{-\frac{\mu \lambda_{i}}{d}}a_{i}, \end{array} \end{array}$$ (7a)

$$\begin{array}{cc} G_{\mu } & =\frac{1}{A^{N}}{\hat{f}}^{*}e^{-\frac{2\mu }{d}\Lambda }\hat{f}=\frac{1}{A^{N}}\sum_{i=0}^{N}e^{-\frac{2\mu \lambda_{i}}{d}}(\sum_{j\in {ℐ}_{i}}|{\hat{f}}_{j}{|}^{2})\;\begin{array}{c} \overset{\text{def}}{=}\;\frac{1}{A^{N}}\sum_{i=0}^{N}e^{-\frac{2\mu \lambda_{i}}{d}}b_{i}, \end{array} \end{array}$$ (7b)

where ${ℐ}_{i}$ (|ℐi|=(Ni)(A−1)i) groups indices associated with the *i*th eigenvalue and


$$\begin{array}{c} a_{i}\;\begin{array}{c} \overset{\text{def}}{=}\;\sum_{j\in {ℐ}_{i}}{\hat{f}}_{j}^{*}{\hat{\nu }}_{j},b_{i}\;\begin{array}{c} \overset{\text{def}}{=}\;\sum_{j\in {ℐ}_{i}}|{\hat{f}}_{j}{|}^{2}. \end{array} \end{array} \end{array}$$ (8)

Because $\lambda_{0}=0$ and $\lambda_{i}>0$ for i≥1, it holds that


$$\begin{array}{c} \lim_{\mu \to \infty }F_{\mu }=a_{0}=\frac{\left(1^{\text{T}}\nu )(1^{\text{T}}f\right)}{A^{N}}=\bar{f},\lim_{\mu \to \infty }G_{\mu }=\frac{b_{0}}{A^{N}}=\frac{(1^{\text{T}}f{)}^{2}}{(A^{N}{)}^{2}}={\bar{f}}^{2}. \end{array}$$ (9)

Thus $b_{0}=A^{N}{\bar{f}}^{2}$ under the Fourier normalisation used here. Expressions written in Fourier-power units subtract *b*_0_, while equivalent expressions written in genotype-vector units subtract $A^{N}{\bar{f}}^{2}$. The operation $P^{*}f$ can be understood as a coordinate transformation and the vector $\hat{f}$ as the landscape f represented in the Fourier domain. The coefficients $b_{0},...,b_{N}$ are obtained from squaring the modulus of Fourier coefficients and therefore represent the power or energy per frequency $\lambda_{i}$. The power spectra of several *NK* landscapes are shown in Fig 7A. For the less rugged landscapes, the power spectrum peaks at a lower frequency than for the more rugged landscapes.

Eqs (7a) and (7b) show that as the accumulated mutations μ increase, the contribution of each Fourier component to the measured fitness decreases at an exponential rate proportional to $\lambda_{i}$, as shown in Fig 7B. For example, for a linear, zero-mean landscape for which only $b_{1}\text{≠}0$, the mean fitness $F_{\mu }$ will decay at a rate proportional to $\lambda_{1}=A$ per accumulated mutation, whereas for a maximally rugged landscape for which only $b_{N}\text{≠}0$, it will decay at a rate proportional to $\lambda_{N}=AN$, which is *N* times faster than the linear one. For most landscapes, the decay will be composed of multiple exponentials, such as for the *NK* landscapes in Fig 7B–7C.

Although the coefficients $a_{0},...,a_{N}$ and $b_{0},...,b_{N}$ could be estimated from Eqs (7a) and (7b), their estimates can become inaccurate (see following section) if $F_{\mu }$ and $G_{\mu }$ are noisy. Assuming that the coefficients $a_{i}$ and $b_{i}$ from Eqs (7a) and (7b) vary significantly in magnitude, the fitness decay curve is dominated by the larger-magnitude exponentials. In the case of a single dominant component, Eqs (5) and (6) can be approximated by $F_{\mu }\approx a_{0}+e^{-\frac{\mu \lambda_{i^{⋆}}}{d}}a_{i^{⋆}}$ and $G_{\mu }\approx (b_{0}+e^{-\frac{2\mu \lambda_{i^{⋆}}}{d}}b_{i^{⋆}})/A^{N}$, where $i^{⋆}$ is the index of the dominant component. We therefore seek to approximate Eq (7a) and (7b) using a single exponential as


$$\begin{array}{cc} F_{\mu } & \approx C_{1}e^{-\mu \rho_{1}}+c_{1}, \end{array}$$ (10a)

$$\begin{array}{cc} G_{\mu } & \approx C_{2}e^{-2\mu \rho_{2}}+c_{2}, \end{array}$$ (10b)

where from Eq (9), it holds that $c_{1}=\bar{f}$ and $c_{2}={\bar{f}}^{2}$. We introduce the decay rates $\rho_{1}$ and $\rho_{2}$ as scalar ruggedness metrics that approximate the average of the frequencies $\lambda_{i}$ weighted by $a_{i}$ or $b_{i}$. This can be shown by expanding the right-hand sides of Eqs (10a) and (10b) as


$$\begin{array}{cc} C_{1}e^{-\mu \rho_{1}}+c_{1} & =C_{1}(1-\mu \rho_{1}+\frac{1}{2}\mu^{2}\rho_{1}^{2}+...)+c_{1}=C_{1}+c_{1}-C_{1}\mu \rho_{1}+𝒪(\mu^{2}), \end{array}$$ (11a)

$$\begin{array}{cc} C_{2}e^{-2\mu \rho_{2}}+c_{2} & =C_{2}(1-2\mu \rho_{2}+2\mu^{2}\rho_{2}^{2}+...)+c_{2}=C_{2}+c_{2}-2C_{2}\mu \rho_{2}+𝒪(\mu^{2}), \end{array}$$ (11b)

and the right-hand sides of Eqs (7a) and (7b) as


$$\begin{array}{cc} \sum_{i=0}^{N}e^{-\frac{\mu \lambda_{i}}{d}}a_{i} & =\sum_{i=0}^{N}a_{i}-\frac{\mu }{d}\sum_{i=0}^{N}a_{i}\lambda_{i}+𝒪(\mu^{2}), \end{array}$$ (12a)

$$\begin{array}{cc} \frac{1}{A^{N}}\sum_{i=0}^{N}e^{-\frac{2\mu \lambda_{i}}{d}}b_{i} & =\frac{1}{A^{N}}\sum_{i=0}^{N}b_{i}-\frac{1}{A^{N}}\frac{2\mu }{d}\sum_{i=0}^{N}b_{i}\lambda_{i}+𝒪(\mu^{2}) \end{array}$$ (12b)

Ignoring higher-order terms, comparing coefficients yields $C_{1}+c_{1}=\sum_{i=0}^{N}a_{i}$ and $C_{1}\mu \rho_{1}=\mu /d\sum_{i=0}^{N}a_{i}\lambda_{i}$ for $F_{\mu }$, and $C_{2}+c_{2}=\sum_{i=0}^{N}b_{i}/A^{N}$ and $C_{2}\mu \rho_{2}=\mu /d\sum_{i=0}^{N}b_{i}\lambda_{i}/A^{N}$ for $G_{\mu }$. We also note that since $\lambda_{0}=0$, Eq (7) and Eqs (9)–(10) imply that $c_{1}=a_{0}=\bar{f}$ for $F_{\mu }$ and $c_{2}=b_{0}/A^{N}={\bar{f}}^{2}$ for $G_{\mu }$, so that


$$\begin{array}{cc} \rho_{1} & \approx \frac{1}{d}\frac{\sum_{i=0}^{N}a_{i}\lambda_{i}}{\sum_{i=0}^{N}a_{i}-a_{0}}=\frac{1}{d}\frac{{\hat{f}}^{*}\Lambda \hat{\nu }}{{\hat{f}}^{*}\hat{\nu }-a_{0}}=\frac{1}{d}\frac{f^{\text{T}}\Delta \nu }{f^{\text{T}}\nu -\bar{f}}, \end{array}$$ (13a)

$$\begin{array}{cc} \rho_{2} & \approx \frac{1}{d}\frac{\sum_{i=0}^{N}b_{i}\lambda_{i}}{\sum_{i=0}^{N}b_{i}-b_{0}}=\frac{1}{d}\frac{{\hat{f}}^{*}\Lambda \hat{f}}{{\hat{f}}^{*}\hat{f}-b_{0}}=\frac{1}{d}\frac{f^{\text{T}}\Delta f}{f^{\text{T}}f-A^{N}{\bar{f}}^{2}}. \end{array}$$ (13b)

The right-hand side of Eq (13b) highlights that the analytical decay rate $\rho_{2}$ for $G_{\mu }$ is the Dirichlet energy $f^{\text{T}}\Delta f$ normalised by $f^{\text{T}}f-A^{N}{\bar{f}}^{2}$, equivalently $f^{\text{T}}f-b_{0}$. Eq (13b) also shows that $\rho_{2}$ is related to other spectral measures such as the *R*^2^ measure, $R^{2}\;\begin{array}{c} \overset{\text{def}}{=}\;\sum_{i=2}^{N}b_{i}/(\sum_{j=1}^{N}b_{j}) \end{array}$, or the spectral entropy, $H\;\begin{array}{c} \overset{\text{def}}{=}\;-\sum_{i=1}^{N}p_{i}\log(p_{i}) \end{array}$, where $p_{i}\;\begin{array}{c} \overset{\text{def}}{=}\;b_{i}/(\sum_{j=1}^{N}b_{j}) \end{array}$. However, note that Eq (13b) explicitly takes the mutation spectrum into account, whereas other measures do not.

From the first-order approximation in Eq (11), the analytical metric $\rho_{2}$ characterises the $G_{\mu }$ decay for small μ. Additionally, Jensen’s inequality for convex functions can be used to show that


$$\begin{array}{c} G_{\mu }=\frac{b_{0}}{A^{N}}+\frac{B}{A^{N}}\sum_{i=1}^{N}\frac{b_{i}}{B}e^{-\frac{2\mu \lambda_{i}}{d}}\geq \frac{b_{0}}{A^{N}}+\frac{B}{A^{N}}e^{-2\mu \rho_{2}}, \end{array}$$ (14)

where $B=\sum_{i=1}^{N}b_{i}$, i.e., the single exponential decaying at rate $\rho_{2}$ provides a lower bound.

We note that $\rho_{1}$ for $F_{\mu }$ depends on the initial population ν, whereas for a sufficiently large number of samples in Eq (6), $\rho_{2}$ for $G_{\mu }$ reflects a global property of the landscape if computed from all starting points. We therefore distinguish between analytical decay rates ($\rho_{1}$, $\rho_{2}$), fitted decay rates ($\rho_{1}^{fit}$, $\rho_{2}^{fit}$), and—for $\rho_{2}$—its local ($\rho_{2}^{loc}$) and global ($\rho_{2}^{glob}$) fitted variants. A summary of the notation used is provided in S1 Table, including the decay rates for biased mutation spectra introduced in the following section. If a single component $a_{i^{⋆}}$ or $b_{i^{⋆}}$ is dominating, then the corresponding decay rates satisfy approximately


$$\begin{array}{c} \rho_{1}\approx \rho_{2}\approx \frac{\lambda_{i^{⋆}}}{d}\leq \frac{A}{A-1}, \end{array}$$ (15)

with fitted decay rates being approximately equal to the one in Eq (15).

The approximation from Eqs (10a) and (10b) can be analysed in more detail for house-of-cards and *NK* landscapes (see Section D of S1 Appendix), where it can be proved that the approximations hold increasingly well in the limit N→∞ [39].

### Biased mutation spectra and landscape perturbations

The single-mutation operator *D* allows the fitness decay to be related to the Hamming graph Laplacian, which is amenable to a Fourier decomposition. In this scenario, all mutations are equally likely, and each population member evolves according to an unbiased random walk on a regular, undirected graph. However, in practice, the mutation spectra are biased, e.g., due to transition/transversion preferences, context effects, or codon usage. Also natural selection, e.g., due to overexpressed proteins, can be modelled as mutation bias. These effects can result in biased walks on weighted, directed or undirected graphs, whose Laplacians are *not* amenable to a Fourier decomposition. Separately, structural perturbations of the fitness landscape, such as the introduction of minimum-fitness genotypes, neutral ridges or modular structures, alter the landscape Fourier spectrum even when the underlying mutation process remains unbiased. We therefore examine both mutation bias and controlled landscape perturbations in the following.

### Biased mutations

Suppose that the graph is weighted but undirected with mutation operator $\tilde{D}$ and symmetric Laplacian $\tilde{\Delta }$, which occurs when, e.g., all transition mutations are preferred over transversion mutations. Since $\tilde{\Delta }={\tilde{\Delta }}^{\text{T}}$, the equations of the previous section remain valid. In particular, the matrix identity from Eq (6), $\exp(-\frac{\mu }{d}\tilde{\Delta })\exp(-\frac{\mu }{d}{\tilde{\Delta }}^{\text{T}})\equiv \exp(-\frac{2\mu }{d}\tilde{\Delta })$ holds, which requires $\tilde{\Delta }$ and ${\tilde{\Delta }}^{\text{T}}$ to commute. However, the analytical squared-decay metric ${\tilde{\rho }}_{2}$ approximates the normalised Dirichlet energy on the weighted graph instead:


$$\begin{array}{c} {\tilde{\rho }}_{2}\approx \frac{1}{d}\frac{f^{\text{T}}\tilde{\Delta }f}{f^{\text{T}}f-{\tilde{b}}_{0}}, \end{array}$$ (16)

where ${\tilde{b}}_{0}=b_{0}=A^{N}{\bar{f}}^{2}$. Since $\tilde{D}$ is symmetric, row-stochastic, and irreducible, the population converges to the uniform distribution, and ${\tilde{G}}_{\mu }$ to the squared uniform landscape average. The metrics $\rho_{2}$ and ${\tilde{\rho }}_{2}$ are associated with particular mutation operators and may differ in general. A landscape may appear rugged via $\rho_{2}$ and smooth via ${\tilde{\rho }}_{2}$. Letting $E_{\tilde{\Delta }}\;\begin{array}{c} \overset{\text{def}}{=}\;\Delta -\tilde{\Delta }=d(\tilde{D}-D) \end{array}$, the difference $\left|\rho_{2}-{\tilde{\rho }}_{2}\right|$ can be bounded as


$$\begin{array}{c} |\rho_{2}-{\tilde{\rho }}_{2}|=\frac{1}{d}\frac{\left|f^{\text{T}}E_{\tilde{\Delta }}f\right|}{\left|f^{\text{T}}f-b_{0}\right|}\leq \frac{1}{d}\frac{\|f{\|}_{2}^{2}\|E_{\tilde{\Delta }}{\|}_{2}}{\left|f^{\text{T}}f-b_{0}\right|}. \end{array}$$ (17)

Thus the metrics are close when the mutation kernel perturbation $\|D-\tilde{D}{\|}_{2}$ is small.

For a directed graph, let the non-symmetric mutation operator and Laplacian be $\bar{D}$ and $\bar{\Delta }$, respectively. Assume that $\bar{D}$ is row-stochastic and irreducible, so that $\bar{D}1=1$, and assume that $\bar{\Delta }=\bar{P}\bar{\Lambda }{\bar{P}}^{-1}$ is diagonalisable with a simple zero eigenvalue and all other eigenvalues having positive real part. This represents a more general configuration, which can occur, e.g., under selection pressure or using *in vivo* mutagenesis methods [40]. In this configuration, the population converges exponentially to its stationary distribution, and ${\bar{G}}_{\mu }$ converges to the square of the stationary-weighted fitness average:


$$\begin{array}{c} {\bar{G}}_{\infty }\;\begin{array}{c} \overset{\text{def}}{=}\;\lim_{\mu \to \infty }{\bar{G}}_{\mu }=\frac{1}{A^{N}}f^{\text{T}}\bar{d}1^{\text{T}}(\bar{d}1^{\text{T}}{)}^{\text{T}}f=(f^{\text{T}}\bar{d}{)}^{2}, \end{array} \end{array}$$ (18)

where ${\bar{d}}^{\text{T}}\bar{D}={\bar{d}}^{\text{T}}$ with ${\bar{d}}^{\text{T}}1=1$ is the stationary distribution induced by the mutation kernel. Without commutativity of $\bar{\Delta }$ and ${\bar{\Delta }}^{\text{T}}$, the expression $exp(-\frac{\mu }{d}{\bar{\Delta }}^{\text{T}})exp(-\frac{\mu }{d}\bar{\Delta })$ cannot be simplified. Instead, consider the first-order approximation $exp(-\frac{\mu }{d}\bar{\Delta })\approx I-\frac{\mu }{d}\bar{\Delta }$, which, when substituted in Eq (6), yields


$$\begin{array}{c} {\bar{G}}_{\mu }\approx \frac{1}{A^{N}}f^{\text{T}}\left(I-\frac{2\mu }{d}\frac{\bar{\Delta }+{\bar{\Delta }}^{\text{T}}}{2}\right)f+𝒪(\mu^{2}), \end{array}$$ (19)

where ${\bar{\Delta }}_{\text{sym}}\;\begin{array}{c} \overset{\text{def}}{=}\;(\bar{\Delta }+{\bar{\Delta }}^{\text{T}})/2 \end{array}$ is the orthogonal projection (via Frobenius norm) of $\bar{\Delta }$ onto the subspace of symmetric matrices. Comparing coefficients with Eq (11) results in the approximation


$$\begin{array}{c} {\bar{\rho }}_{2}\approx \frac{1}{d}\frac{f^{\text{T}}{\bar{\Delta }}_{\text{sym}}f}{f^{\text{T}}f-{\bar{b}}_{0}}, \end{array}$$ (20)

where ${\bar{b}}_{0}=A^{N}(f^{\text{T}}\bar{d}{)}^{2}$ is the weighted, squared landscape average. As for the weighted, undirected setting, the difference $\left|\rho_{2}-{\bar{\rho }}_{2}\right|$ may be upper-bounded using the perturbation $E_{\bar{\Delta }}\;\begin{array}{c} \overset{\text{def}}{=}\;\Delta -{\bar{\Delta }}_{\text{sym}} \end{array}$ as


$$\begin{array}{c} |\rho_{2}-{\bar{\rho }}_{2}|\leq \frac{1}{d}\left(\frac{\|f{\|}_{2}^{2}\|E_{\bar{\Delta }}{\|}_{2}}{\left|f^{\text{T}}f-b_{0}\right|}+\frac{\left|f^{\text{T}}{\bar{\Delta }}_{\text{sym}}f||b_{0}-{\bar{b}}_{0}\right|}{\left|f^{\text{T}}f-b_{0}||f^{\text{T}}f-{\bar{b}}_{0}\right|}\right), \end{array}$$ (21)

but the bound now also contains a term proportional to the difference between the uniform and stationary-weighted asymptotes.

**Fig 6:**
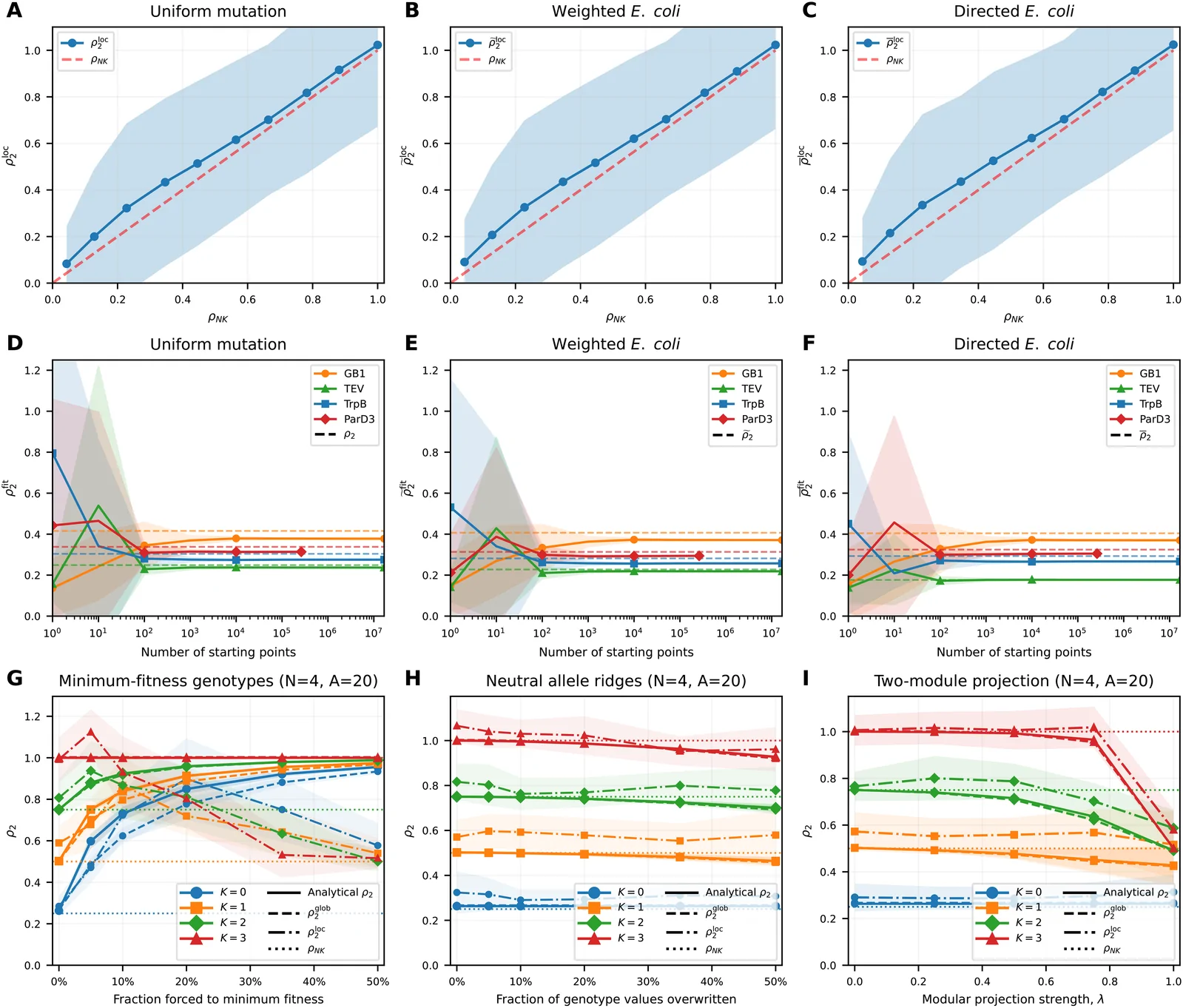
Ruggedness inference under mutation bias and landscape perturbations. **A–C:** Local decay-rate estimates for nucleotide-encoded *NK* landscapes under uniform **(A)**, weighted-undirected **(B)**, and directed *E. coli* (**C**) mutation kernels. The analysis used a representative set of 160,000 codon sequences for each landscape. Points and shaded regions show the mean and SD across landscapes and starting populations. **D–F:** Global decay-rate estimates for the codon-expanded GB1, TrpB, TEV, and ParD3 landscapes under the corresponding three mutation kernels. All nucleotide genotypes were included. Solid lines and shaded regions show the mean and SD of bootstrap estimates as the number of starting genotypes increases; dashed lines denote the corresponding analytical metrics. **G–I:** Effects of increasing fractions of minimum-fitness genotypes **(G)**, neutral ridges **(H)**, and modular projection strength (**I**) on *N* = 4, *A* = 20 *NK* landscapes. Solid, dashed, and dash-dotted lines denote analytical $\rho_{2}$, fitted $\rho_{2}^{glob}$, and fitted $\rho_{2}^{loc}$, respectively; shaded regions show the SD across 20 landscapes. Simulation parameters and further details are provided in Section G of S1 Appendix.

To qualitatively assess the impact of biased mutation, the ruggedness analysis from Fig 3B was repeated in Fig 6A–C for different nucleotide-level mutation kernels. The amino-acid *NK* landscapes were represented at the nucleotide level using a codon-to-amino-acid mapping, and the analysis was performed on a representative set of 160,000 codon sequences. Fig 6A uses a uniform mutation kernel, $D=(11^{\text{T}}-I)/3$, whereas Fig 6C uses a directed, row-stochastic *E. coli* kernel $\bar{D}$ adapted from [41]. Fig 6B uses an undirected, symmetric kernel $\tilde{D}=Q((\bar{D}+{\bar{D}}^{\text{T}})/2)Q$, where the diagonal matrix *Q* is obtained by symmetric Sinkhorn scaling such that $\tilde{D}$ is symmetric and doubly stochastic. Fig 6A–C show that the mutation kernels considered have no major effect on ruggedness estimation for *NK* landscapes. The estimates are instead dominated by the homogeneous statistical structure of the *NK* model.

Fig 6D–6F extend this analysis to empirical landscapes by repeating the population-sensitivity simulations from Fig 4D for the three mutation kernels used in Fig 6A–6C. In contrast to the representative sampling used for the *NK* landscapes, these simulations were performed over all 4^3*N*^ genotypes. The figures show the fitted estimates $\rho_{2}^{fit}$ as a function of the number of starting points (solid lines), with shaded regions indicating the SD and dashed lines denoting the corresponding analytical squared-decay metrics $\rho_{2}$ (**D**), ${\tilde{\rho }}_{2}$ (**E**), and ${\bar{\rho }}_{2}$ (**F**). The nucleotide-to-amino-acid mapping spreads the landscape spectra across a broader range of frequencies and generally produces a small reduction in the inferred ruggedness. Consequently, fewer starting points are required to obtain a stable global estimate (cf. Fig 4B). However, because the resulting ruggedness values are more closely spaced, greater estimation accuracy is needed to reliably distinguish between ruggedness levels. S2 Fig repeats the analysis using a mutation model for *A. thaliana* and shows the same qualitative behaviour, and all decay rates are summarised in S2 Table. Further details are provided in Section G of S1 Appendix.

### Landscape perturbations

We assessed whether the decay-based ruggedness estimate remains informative when the fitness landscape is subjected to controlled structural perturbations while the unbiased mutation process is retained (Fig 6G–6I). We generated synthetic *NK* landscapes (*N* = 4, *A* = 20, K∈{0,1,2,3}, with 20 landscapes per value of *K*) and modified each landscape in three ways. In Fig 6G, an increasing fraction of randomly selected genotypes was assigned the minimum fitness of the corresponding landscape, thereby creating progressively larger minimum-fitness regions. In Fig 6H, neutral ridges were introduced by selecting one site and copying the fitness slice associated with a reference allele to an increasing number of alternative allelic slices at that site, making the corresponding regions identical in fitness. In Fig 6I, modularity was introduced by interpolating between the original landscape f and a two-module landscape, $f_{mod}\;\begin{array}{c} \overset{\text{def}}{=}\;𝔼[f|x_{1},x_{2}]+𝔼[f|x_{3},x_{4}]-𝔼[f] \end{array}$, such that $f_{\lambda }\;\begin{array}{c} \overset{\text{def}}{=}\;(1-\lambda )f+\lambda f_{mod} \end{array}$, with λ∈[0,1]. This construction preserves interactions within each two-site module while removing interactions between the two modules. For all three perturbations, a perturbation strength of zero corresponds to the original *NK* landscape. The analytical value of $\rho_{2}$ (solid lines) was recomputed directly from the Fourier spectrum of each perturbed landscape, whereas $\rho_{2}^{glob}$ (dashed lines) and $\rho_{2}^{loc}$ (dash-dotted lines) were estimated from simulated fitness trajectories on the same landscape.

Fig 6G shows that increasing the fraction of minimum-fitness genotypes raises $\rho_{2}$, consistent with the introduction of sharp fitness discontinuities that redistribute spectral power towards higher frequencies. This effect is stronger for less rugged landscapes than for rugged landscapes, which already contain substantial high-frequency power. Conversely, Fig 6H shows that increasing the extent of the neutral ridges reduces $\rho_{2}$, because copying fitness slices suppresses variation along the affected directions and shifts spectral power towards lower frequencies. This effect is more pronounced for rugged landscapes than for less rugged landscapes, whose spectra are already dominated by lower-frequency components. Finally, Fig 6I shows that increasing the modular projection strength λ reduces $\rho_{2}$. In particular, $\rho_{2}$ decreases from 1.0 to 0.5 for *K* = 3 and from 0.75 to 0.491 for *K* = 2, reflecting the fact that, at λ=1, no interaction can involve more than two sites.

Fig 6G–6I further show that the global estimates, $\rho_{2}^{glob}$, consistently reproduce the analytical trends. The local estimates, $\rho_{2}^{loc}$, also reproduce the trends for the neutral-ridge and modularity perturbations in Fig 6H and 6I, but not for the minimum-fitness perturbation in Fig 6G, which produces increasingly heterogeneous landscapes as the fraction of minimum-fitness genotypes grows.

### Estimating power spectra and ruggedness parameters

**Fig 7:**
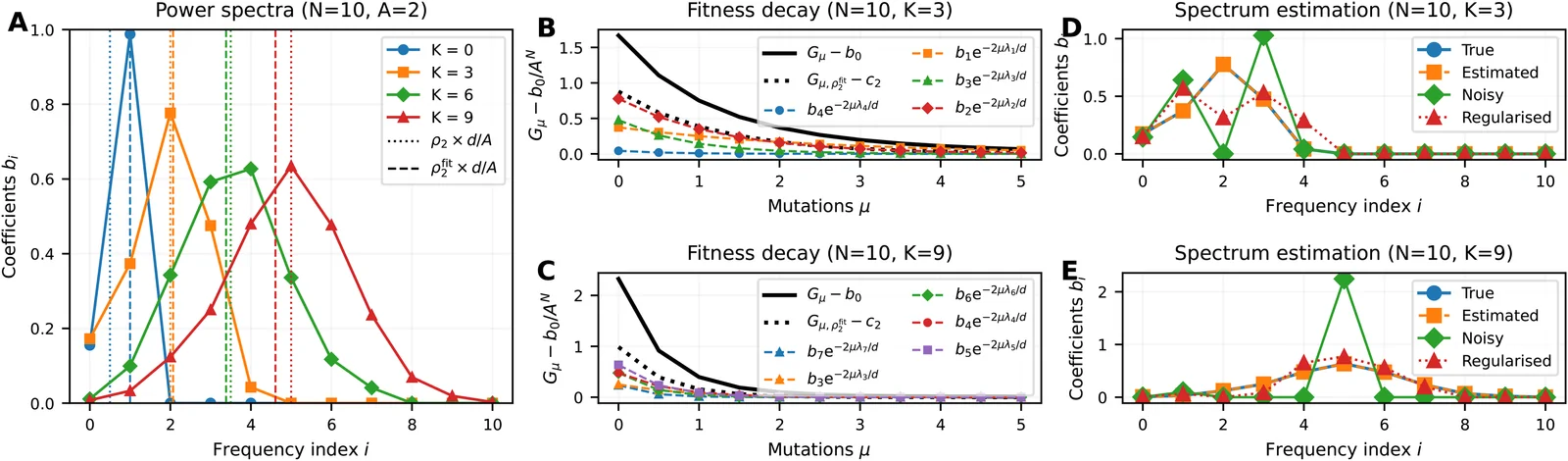
Power-spectrum estimation on *NK* landscapes (*N* = 10, *A* = 2). **A:** Power spectra for K∈{0,3,6,9}. **B–C:** Fitness-decay curves for *K* = 3 and *K* = 9, respectively. Shown are the non-constant decay $G_{\mu }-b_{0}/A^{N}$, the single-rate fit $G_{\mu ,\rho_{2}^{fit}}-c_{2}$ from Eq (26), and the largest non-constant components $b_{i}e^{-2\mu \lambda_{i}/d}/A^{N}$ of the decay decomposition. **D–E:** Power-spectrum estimates for *K* = 3 and *K* = 9, respectively. The noiseless estimate uses constrained least squares (Eq (22)); noisy estimates use non-negative least squares with or without regularisation (Eq (24)). Regularisation reduces the effect of noise.

Generally, the power spectrum of a fitness landscape is composed of both low-frequency and high-frequency terms, as shown in Fig 7A. Practically, if the landscape *f* is known, its power spectrum can be computed by reshaping *f* as an *N*-dimensional cube of size *A* along each axis, applying an *N*-dimensional Fast Fourier Transform, and grouping the terms as in Eq (7b).

If the landscape is not known, the power spectral coefficients $b_{i}$ from Eq (8) can be estimated using the decay curve $G_{\mu }$ from Eq (7b). The procedure for estimating $a_{i}$ from $F_{\mu }$ is similar. Let $G=(G_{0},G_{\mu_{1}},...,G_{\mu_{M}}{)}^{\text{T}}$ be the squared average fitness measurements of populations accumulating $0,\mu_{1},...,\mu_{M}$ mutations and define $b=(b_{0},...,b_{N}{)}^{\text{T}}$. The following least squares problem can be used to estimate *b* from fitness measurements *G*:


$$\begin{array}{c} b=\underset{x\in {\mathbb{R}}_{\geq 0}^{N+1}}{\text{arg min}}\|G-\Phi x{\|}_{2}^{2}, \end{array}$$ (22)

where element *i*,*j* of the matrix $\Phi \in {\mathbb{R}}^{\left(M+1)\times (N+1\right)}$ is defined as


$$\begin{array}{c} \Phi_{i,j}=e^{-\frac{2\mu_{i}\lambda_{j}}{d}}, \end{array}$$ (23)

for i=0,...,M and j=0,...,N. For noise-free observations, problem (22) can be solved using standard techniques to accurately determine the fitness landscape spectrum *b* (Fig 7D–7E). However, the addition of noise with a signal-to-noise ratio ≤10 can already significantly perturb the estimate from Eq (22), because the ill-conditioned Φ amplifies the estimation error. To improve the robustness of the estimate, Eq (22) can be extended with a regularisation term as


$$\begin{array}{c} b=\underset{x\in {\mathbb{R}}_{\geq 0}^{N+1}}{\text{arg min}}\|G-\Phi x{\|}_{2}^{2}+\gamma \|x{\|}_{2}^{2}, \end{array}$$ (24)

where γ≥0 is a regularisation parameter. While the results from Fig 7D–7E show that regularisation can improve the estimation accuracy, it depends on having chosen γ appropriately; choosing a poor γ will lower accuracy. The inference method could be developed further, incorporating more knowledge about the distribution of *b* and the noise process, which is beyond the scope of this paper.

Instead of estimating the full spectrum, we estimate fitted ruggedness parameters using the approximations from Eq (10). The $\rho_{2}^{fit}$ obtained from fitting $G_{\mu }$ will in general not equal the analytical $\rho_{2}$ from Eq (13b); according to Eq (14), the analytical $\rho_{2}$ defines a lower-bound single exponential. If the power spectrum is concentrated in a single frequency (such as for *NK* landscapes), then $\rho_{2}^{fit}\approx \rho_{2}$. The scalar parameters *C*_2_, $\rho_{2}^{fit}$, and *c*_2_ are unknown *a priori* and must be estimated from data. As for the power spectrum estimation, the squared average fitness values *G* are recorded for a population accumulating $\mu_{1},...,\mu_{M}$ mutations. We then seek to find *C*_2_, $\rho_{2}^{fit}$, and *c*_2_ such that


$$\begin{array}{c} G_{\mu_{i}}\approx C_{2}e^{-2\mu_{i}\rho_{2}^{fit}}+c_{2}. \end{array}$$ (25)

As for the full spectrum estimation, the mutations are stochastic and the decay curves are noisy, which impacts the parameter estimation process. To simplify the estimation, we assume that *G*_0_ is noise-free, and that the curve fitted via Eq (25) must pass through it, which allows us to discard parameter *C*_2_ by setting $C_{2}=G_{0}-c_{2}$. For the remaining data points, we seek to find $\rho_{2}^{fit}$ and *c*_2_ that minimise the sum of squares error:


$$\begin{array}{c} \min_{\rho_{2}^{fit},c_{2}\in {\mathbb{R}}_{\geq 0}}\sum_{i=1}^{M}{\left((G_{0}-c_{2})e^{-2\mu_{i}\rho_{2}^{fit}}-(G_{\mu_{i}}-c_{2})\right)}^{2}. \end{array}$$ (26)

The procedure for estimating $\rho_{1}^{fit}$ from $F_{\mu }$ is similar and requires discarding the factor 2 from exponentials. As long as $c_{2}\text{≠}0$, Eq (26) is a nonlinear optimisation problem that cannot be solved using standard least squares techniques and may have multiple local optima. Here, we are using standard scientific packages for constrained nonlinear optimisation (specifically, the curve_fit function from the scipy.optimize package [42]), which returns a solution within 1 s on a standard laptop. Initialising the routine with $\rho_{2}^{fit}{|}_{\text{init}}=0.5$ and $c_{2}{|}_{\text{init}}=G_{M}$ and constraining the optimisation variables by $\rho_{2}^{fit}\in [0,A/(A-1)]$ and $c_{2}\in [0.5\times c_{2}{|}_{\text{init}},1.5\times c_{2}{|}_{\text{init}}]$ (see Code Availability), this procedure is robust to noise and could be extended with uncertainty weights (regularisation). The curve fitting for two *NK* landscapes is shown in Fig 7B–7C, with the fitted decay rates $\rho_{2}^{fit}$ shown in Fig 7A. For these examples, the power spectrum peaks at a particular frequency, so that Eq (25) results in a good approximation of the true decay composed of multiple exponentials.

## Supporting information

S1 Appendix Supplementary methods and analyses. Contains additional details on mutagenesis and directed-evolution simulations, alternative ruggedness metrics, special cases of the decay-rate metric, empirical fitness landscapes, alternative landscape models, and alternative mutation models [2,4,6–8,10,24–26,39,41,43,44].
(PDF)

S1 TableSummary of ruggedness and decay-rate notation.Definitions of the analytical and fitted metrics derived from the average fitness decay $F_{\mu }$ and squared average fitness decay $G_{\mu }$.(PDF)

S2 Table Analytical decay rates for empirical landscapes. Decay-rate metrics for the GB1, TrpB, TEV, and ParD3 landscapes under uniform, weighted-undirected, and directed mutation models.
(PDF)

S1 Fig Comparison of strategy spaces and decay curves across landscape models. Strategy spaces and corresponding fitness-decay curves for *NK*, Rough Mount Fuji, and stochastic block-model landscapes of varying ruggedness.
(PDF)

S2 Fig Ruggedness inference using an *A. thaliana* mutation model. Replication of Fig 6A-F using weighted-undirected and directed *A. thaliana* mutation kernels on synthetic *NK* and empirical landscapes.
(PDF)

S3 Fig Decay curves on empirical landscapes under different mutation models. Sampled, fitted, and analytical squared-fitness decay curves for empirical landscapes under uniform, weighted-undirected, and directed mutation kernels.
(PDF)

S4 Fig Strategy look-up tables for longer directed-evolution experiments. Optimal base-chance and population-splitting parameters for *NK* landscapes over 25, 50, and 100 directed-evolution iterations.
(PDF)
