# Supplementary material for: Sequence-free landscape inference for directed evolution
Source: PLoS Comput Biol. 2026 Sep 3;22(9):e1014713. doi: 10.1371/journal.pcbi.1014713 (PMC13585340; doi:10.1371/journal.pcbi.1014713)
Supplement: Supplementary file 1 [file pcbi.1014713.s001.pdf]

S1 Appendix for *Sequence-free Landscape Inference for Directed Evolution*

A Simulating mutagenesis and selection

In our simulations, we represent a population of P cells at iteration k as $\boldsymbol{p}_k = (\boldsymbol{x}_k^1, \ldots, \boldsymbol{x}_k^P)$, where $\boldsymbol{x}_k^i \in \mathcal{G}$ is the genotype of cell i. Given an empirical or theoretical landscape f, each genotype is associated with a fitness $f_k^i = f(\boldsymbol{x}_k^i)$. When the population $\boldsymbol{p}_k$ is mutagenised, each member $\boldsymbol{x}_k^i$ is exposed to X single mutations to yield $\boldsymbol{x}_{k+1}^i$, where X is an independent and identically distributed Poisson random variable. To obtain the decay curves used for ruggedness parameter estimation, we repeat the mutagenesis process for M iterations and record the average population fitness $F_k = 1/P \sum_{i=1}^{P} f_k^i$, the squared mean fitness F_k^2, which is subsequently averaged across starting populations to estimate G_μ, and the average accumulated mutations μ_k. This process is repeated and averaged over different starting populations $\boldsymbol{p}_0$ to generate a decay curve such as the one shown in Fig 3A.

For the selection process used in directed evolution (DE) experiments, each iteration is extended with a selection procedure. Here, we use an approach developed in [10] that parameterises selection using probabilistic selection functions. The population members $\boldsymbol{x}_k^i$ are ranked according to their fitness f_k^i. A fraction of cells is then randomly selected from $\boldsymbol{p}_k$ according to the probability distribution shown in Fig 1 (top right). This distribution is parameterised by two parameters: the base chance b and threshold t. If a cell's fitness f_k^i is above the rank threshold t (within the population $\boldsymbol{p}_k$), it is guaranteed to be selected for iteration $k+1$. Otherwise, the selection probability is b, which is independent between cells. Here, the fraction of cells selected at each iteration is kept constant as $\mathrm{f} = 0.2$, fixing t as $t = (1 - \mathrm{f})/(1 - b)$. Each cell that is not selected is replaced by a new cell with genotype taken at random (uniformly and independently) from the pool of cells that were selected. For example, for $t = 0.8$ and $b = 0$ the top $20\,\%$ of fittest cells are selected for the next iteration. The case $t = 1.0$ and $b = 0.2$ represents a selection-free scenario, with a random $20\,\%$ of cells surviving each generation. All simulations were carried out using a GPU-based DE package published in [10].

B Simulating directed evolution

For simulating DE experiments, the selection and mutagenesis procedures are repeatedly applied to a starting population $\boldsymbol{p}_0$. The selection function is parameterised by the base chance b, while the rank threshold t is fixed via $t = (1 - \mathrm{f})/(1 - b)$. The fraction of selected cells f is set to $\mathrm{f} = 0.2$, so that at each iteration, approximately $20\,\%$ of the population is selected for the following iteration. The value of b is determined using the decay rate estimate ρ^{fit}. Before starting DE, the population $\boldsymbol{p}_0$ is repeatedly mutagenised without selection for M iterations. The resulting decay curve is used to estimate ρ^{fit}, which is in turn used to select b from a pre-computed map that connects ρ_{NK} grid

values to optimal selection parameters. This map is pre-computed according to the work presented in [10]. In addition to the parameter b, the work from [10] also incorporates a population splitting parameter s that subdivides the population $\boldsymbol{p}_0$ into s smaller populations, yielding s smaller DE experiments that run in parallel. This is illustrated in Fig 1 (bottom right).

The map linking NK ruggedness to the optimal parameter set (b_ρ, s_ρ) is computed using NK landscapes, whose grid values are $\rho_{NK} = (K+1)/N$ via Eq (S2). For fixed N and A, we generate a number of random NK landscapes for $K = 1, \dots, N-1$. For each K, we run DE experiments for different starting populations $\boldsymbol{p}_0$ and different parameter sets (b, s), and store best-performing parameters for each K, defining a map from (N, K) to (b, s). Given N and the fitted estimate ρ^{fit}, the parameter set associated with the value of ρ_{NK} closest to ρ^{fit} is selected. An example is shown in Fig 1 (middle) for $N = 4$, $A = 20$, and $K \in \{1, 2, 3\}$ ($\rho_{NK} \in \{0.5, 0.75, 1\}$). Note that while ρ^{fit} can take on continuous positive values, ρ_{NK} can only take on discrete values for fixed N. Alternatively, the generated maps can be averaged across different values of N.

C Other ruggedness metrics

Various measures have been proposed to quantify landscape ruggedness, most of which require both genotypic and phenotypic information. For our analysis, we compare the analytical decay-rate measure ρ_2 with six other measures, summarised below. Most metrics were originally defined on an allele set of size 2 ($|\mathcal{A}| = 2$), and are extended here to larger sets ($|\mathcal{A}| > 2$). For a more comprehensive discussion of ruggedness measures, we refer the reader to [8].

Roughness to slope ratio (r/s):

Given a genotype $\boldsymbol{x} = (\sigma_1, \dots, \sigma_N)^\intercal \in \mathcal{G}$, a landscape $f_{lin} : \mathcal{G} \to \mathbb{R}$ is *linear* if the contribution of each locus and allele is additive, i.e. $f_{lin}(\boldsymbol{x})$ can be written as $f_{lin}(\boldsymbol{x}) = \sum_{i=1}^{N} \phi_i(\sigma_i)$, where $\phi_i : \mathcal{A} \to \mathbb{R}$ is the fitness contribution of locus i for allele σ_i. The roughness to slope ratio [4] captures how well a given landscape f can be approximated by a linear one: After finding the best linear fit f_{lin} to f, i.e. the $N \times A$ values $\phi_i(\sigma)$, the mean slope is defined as $s \stackrel{\text{def}}{=} \sum_{i=1}^{N} \sum_{\sigma_i \in \mathcal{A}} |\phi_i(\sigma_i)|/(AN)$, the roughness as $r \stackrel{\text{def}}{=} (\sum_{x \in \mathcal{G}} (f(\boldsymbol{x}) - f_{lin}(\boldsymbol{x}))^2 / A^N)^{1/2}$, and the roughness to slope ratio as r/s. For a linear landscape, $r/s = 0$, whereas for more rugged landscapes r/s increases.

Landscape R^2 measure (R^2):

The landscape R^2 measure was originally defined in [8] using landscape Fourier analysis and is directly related to the measure ρ_2 introduced in this work. Suppose that the landscape is represented in vector notation as $\boldsymbol{f} \in \mathbb{R}^{A^N}$ and mapped to the Fourier domain as $\hat{\boldsymbol{f}} = P^* \boldsymbol{f}$. Define $b_i \stackrel{\text{def}}{=} \sum_{j \in \mathcal{I}_i} |\hat{\boldsymbol{f}}_j|^2$, where $\mathcal{I}_i$ refers to the indices of $\hat{\boldsymbol{f}}$ associated with the ith eigenvalue of the adjacency matrix. The R^2 measure is then defined as $R^2 \stackrel{\text{def}}{=} (\sum_{i=2}^{N} b_i)/(\sum_{i=1}^{N} b_i) \in [0, 1]$, i.e., the higher-order epistasis frac-

tion. For a linear landscape, $R^2 = 0$, whereas for a more rugged landscape R^2 increases. The R^2 measure can be computed from decay curves if the full spectrum is estimated via Eqs (22) or (24). However, the difficulties in estimating the full spectrum can make the R^2 measure less robust than the fitted decay rate ρ_2^{fit}. We denote this quantity as "R^2" because it is equivalent to the coefficient of determination.

Spectral entropy (H):

Similarly to the R^2 measure, the spectral or Shannon entropy H uses the power spectral coefficients b_i and is defined as $H \stackrel{\text{def}}{=} -\sum_{i=1}^{N} p_i \log p_i / \log N \in [0,1]$ with $p_i \stackrel{\text{def}}{=} b_i / \sum_{j=1}^{N} b_j$. The entropy is large when the power is spread uniformly across frequencies, reaching its maximum at 1 when $b_i = b_j \forall i, j \in \{1, \ldots, N\}$, and small if the power is concentrated in a few coefficients, reaching its minimum when $b_j = 0 \forall j \neq i$ for some i.

Local epistasis (n_ϵ):

Epistasis occurs when the fitness contribution of one of the N loci depends on the allele set at other loci, or in terms of mutations, when the effect of one mutation depends on the presence of others. Simple sign epistasis occurs when a mutation is beneficial in the presence of one set of mutations but disadvantageous in another. For example, consider a landscape with $N = 2$ and $\mathcal{A} = \{0, 1\}$, so that $\boldsymbol{x} = (\sigma_1, \sigma_2)^\top$ with $\sigma_i \in \mathcal{A}$. Simple sign epistasis occurs if changing σ_1 from 0 to 1 has a positive effect on fitness when $\sigma_2 = 0$ and a negative effect when $\sigma_2 = 1$. Here, we measure local epistasis as the number of genotype pairs with a Hamming distance 2 that exhibit simple sign epistasis.

Distance to local maxima (d_{max}):

A local maximum is defined as a genotype with higher fitness than all its neighbours. The metric d_{max} measures [6] the Hamming distance to the closest local maximum, averaged over all genotypes. A large d_{max} indicates fewer local maxima, hence a smoother landscape.

Number of direct paths to global maximum (π_{max}):

Given a global fitness maximum at genotype $\boldsymbol{x}^\star$, this metric [7] counts the number of fitness-increasing direct (length N) paths π_{max} from the antipodal genotype $\bar{\boldsymbol{x}}^\star$ to $\boldsymbol{x}^\star$. The antipodal genotype $\bar{\boldsymbol{x}}^\star$ is the genotype where, for each locus, the corresponding alleles in $\bar{\boldsymbol{x}}^\star$ and $\boldsymbol{x}^\star$ are different. For $|\mathcal{A}| > 2$, there are multiple antipodal genotypes, so we define π_{max} to be the mean over all $(A-1)^N$ possible $\bar{\boldsymbol{x}}^\star$. A small π_{max} indicates a more rugged landscape.

D Special cases for decay rate metric

House-of-cards landscapes:

For zero-mean house-of-cards landscapes, the fitness values are assumed to be random, independent, and normally distributed as $\boldsymbol{f}_i \sim \mathcal{N}(0,1)$. Due to the orthonormality of $\boldsymbol{P}$, this results in all $\hat{\boldsymbol{f}}_i$ being independent of one another, and $\hat{\boldsymbol{f}}_i \sim \mathcal{N}(0,1)$, i.e. the Fourier representation inherits the same distribution. As a result, the terms b_i in Eq (7b) are chi-squared distributed with mean equal to the multiplicity of λ_i. The eigenvalue multiplicity $\binom{N}{i}(A-1)^i$ is maximised for $i^\star = \text{round}(N(A-1)/A)$, so that $|b_{i^\star}| \gg |b_i|$ for $i \neq i^\star$. To see why this is true, note that the function $\binom{N}{i}(A-1)^i/A^N$ can be interpreted as a binomial distribution, in which case $|i - i_c^\star|/N \leq 3\sqrt{A-1}/(\sqrt{N}A)$ with $i_c^\star = N(A-1)/A$ contains $\sim 99.7\,\%$ of its weight. Substituting the eigenvalue $\lambda_{i^\star} = Ai^\star$ yields the decay rate for house-of-cards landscapes:

$$\rho_{\text{HoC}} = \frac{1}{d} \times \lambda_{i^\star} = \frac{1}{N(A-1)} \times A\frac{N(A-1)}{A} = 1. \tag{S1}$$

Eq (S1) could also be derived differently. Since every fitness value is independent, with an average fitness of 0, every time a cell mutates its new fitness is on average 0. Hence the fitness curve measures how many non-mutated cells are in the population, which is equal to $\mathrm{e}^{-\mu}$.

NK landscapes:

This analysis can be extended to NK landscapes, which can be viewed as a sum of N smaller house-of-cards landscapes of (effective) dimensionality $K+1$ [39]. For each of the smaller landscapes, the eigenvalue multiplicity is maximised for $i^\star = \text{round}((K+1)(A-1)/A)$, which, when substituted in Eq (13), yields

$$\rho_1 \approx \rho_2 \approx \frac{1}{N(A-1)} \times A\frac{(K+1)(A-1)}{A} = \frac{K+1}{N} \stackrel{\text{def}}{=} \rho_{NK}. \tag{S2}$$

Since for NK landscapes the parameter $K \leq N-1$ measures the number of epistatic interactions and therefore ruggedness, Eq (S2) shows that the decay rate definition of ruggedness is closely related to the NK notion of ruggedness. Specifically, the analytical decay-rate metric satisfies $\rho_1 \approx \rho_2 \approx \rho_{NK}$ for these landscapes.

E Empirical landscapes and ruggedness

To interpret the quantitative predictions of each ruggedness metric it is necessary to consider two factors: the intrinsic properties of the protein and the assay used to generate fitness scores. While we speculate here on differences between landscapes and potential causes for their relative ruggedness, we caution that such interpretations require validation through deeper analysis and complementary measurements to disentangle biological from assay-based contributions.

TEV:

The data for the TEV landscape was downloaded from [26], and converted into a $20{\times}20{\times}20{\times}20$ numpy array. The TEV protease (the most rugged in all metrics except when measured by the spectral entropy H) recognises a highly specific substrate sequence, and its activity depends acutely on maintenance of its catalytic triad [43], as well as on its substrate-binding pocket and global fold. Catalytic triads in particular are among the least mutation-tolerant motifs in biology—even single substitutions can abolish function—which may contribute to the highly rugged landscape observed. The assay used to collect this data was based on DNA recording with a base editor [26]. This data is inherently noisy due to the added layer of biological complexity, which is likely to contribute significantly to the ruggedness of this landscape. However, of note is that this assay is much more linearly proportional to activity than the growth-based assays used to measure TrpB and ParD3.

ParD3:

The data for the ParD3 landscape was downloaded from [24], and converted into a $20 \times 20 \times 20$ numpy array. ParD3 is an antitoxin in which the C-terminal region is intrinsically disordered in the absence of its toxin partner. Such disordered regions typically rely on general physicochemical properties rather than rigid 3D structures, allowing greater mutational tolerance and producing a smoother landscape [44]. From the assay perspective, this fitness landscape was mapped using a growth competition assay [24]. Such an assay is inherently less noisy than that used to evaluate TEV, due to the fact that the measurement is population-level, and the exponential nature of growth also acts to amplify signal at the upper end of fitness. Similarly, growth-based assays of this kind may exhibit “saturation” in the sense that ParD3 variants with toxin binding ability above some threshold at which they can sequester the vast majority of toxin in a cell can thus yield similar (growth-based) fitness scores, even if *in practice* their binding affinity (if it were directly measured) differs significantly. This factor, together with the noise-smoothing properties of this assay, may also contribute to the smoothness of the ParD3 landscape.

TrpB:

The data for the TrpB landscape was downloaded from [25], and converted into a $20 \times 20 \times 20 \times 20$ numpy array. Similarly to ParD3, TrpB was evaluated using a growth-based assay [25], allowing us to more confidently compare the two on a biological basis. TrpB is a subunit of tryptophan synthase, which catalyses the synthesis of the amino acid tryptophan. Unlike ParD3, it does not contain any large, intrinsically disordered regions, and thus many residues may be coupled to maintain the folded structure and activity, contributing to epistasis and therefore landscape ruggedness. Meanwhile, in the case of TrpB this growth coupling is achieved through tryptophan synthesis which, following a similar argument to above, may “saturate” in its impact on measured fitness for

high-performing TrpB variants (i.e. once an adequate quantity of tryptophan is produced in the cell, further increase in enzyme efficiency is not rewarded by this assay).

GB1:

The data for the GB1 landscape was downloaded from [2], and converted into a $20 \times 20 \times 20 \times 20$ `numpy` array. GB1 is an immunoglobulin binding protein, whose structure, similarly to TrpB, is globular. Therefore it must also maintain the amino acid interactions that help support folded structure and activity, and consequently contribute to landscape ruggedness. The fitness values in this case were measured using a high-throughput binding assay [2]. The assay achieved a coverage of approximately 100 reads per variant, helping to reduce the noise of the data and therefore assay-related ruggedness, particularly compared to the TEV example.

Given the above arguments, we believe that the ordering of these landscapes by ρ_2 (Fig 4G) is justifiable. The assay used to measure TEV fitness was inherently noisy, alongside the fact that the presence of a catalytic triad in the enzyme could add considerable interaction effects and therefore ruggedness. For this reason, it makes sense that it would be ranked as more rugged than the remaining landscapes, rather than grouped with them—and indeed *all* ruggedness metrics investigated, except for the spectral entropy H, agreed with this conclusion. GB1 and TrpB, both globular proteins that were measured with less noisy assays, have justification to be grouped together (as they were by ρ_2, as well as roughness to slope and landscape R^2) and similarly to be below TEV. ParD3 was positioned at the bottom by all metrics, which aligns with our expectations given both its intrinsically disordered structure and the properties of the growth-based assay used to define its fitness.

Considering again Fig 4G and the metric comparisons in Fig 3F, we can see significant qualitative differences between metrics. In particular, from Fig 4G, the metrics considered can broadly be classified into three groups: Group 1 (decay rate ρ_2, roughness to slope, and landscape R^2) places TEV as the most rugged, ParD3 as the least, and then groups GB1 and TrpB close together at intermediate ruggedness. Group 2 (local epistasis and distance to local max) places ParD3 at the bottom, GB1 at intermediate ruggedness, but then groups TrpB with TEV at the ruggedness maximum. Group 3 (paths to the global maximum) in turn places TEV, GB1, and TrpB at the scale's maximum ruggedness—perhaps not surprising because this metric (as shown in Fig 3F) saturates very quickly as a landscape becomes more rugged. Considering the biological reasoning above we believe our metric (or more broadly those in Group 1) most aligns with the desirable properties of a ruggedness metric (i.e. as a measure of how sensitive a protein's fitness is to mutation): TrpB, whose growth-based measurements are comparatively smooth, falls below TEV, as does GB1. It remains unclear whether GB1 and TrpB *should* exhibit a similar ruggedness value (as seen for all Group 1 metrics)—this would best be answered with further experimental measurements or aug-

mentation of our comparisons with additional protein landscapes as they become available in the future.

F Alternative landscape models

In order to explore the effects of other landscape models on the behaviour of SLIDE, a Rough Mount Fuji model and a stochastic block model were implemented (S1 Fig). For each model, the optimal strategy choice across landscapes of low, mid and high ruggedness was compared. The optimum strategy for all models shifts from low base chance and low splitting to high base chance and high splitting as ruggedness increases, supporting the transferability of SLIDE across landscape models. In addition, the results also confirm that the expected trend in fitness decay curves holds across the different models, with higher ruggedness landscapes exhibiting higher rates of fitness decay in all cases. These results suggest that SLIDE is not specific to the NK model and general trends should hold across other landscape models and empirical landscapes.

G Alternative mutation models

While this work generally assumes an unbiased mutation spectrum, the assumption that transitions between all 20 amino acids are equally probable is biologically unrealistic. For the simulations described in the Biased Mutation Spectra and Landscape Perturbations section, the mutation model is extended. First, for Fig 6A-6C, the model is extended with codon effects, mapping each nucleotide triplet to a codon encoding a specific amino acid or a stop signal. To incorporate this, a landscape of N amino acids is represented by $3N$ nucleotides. We then use codon mapping to translate these sequences back into the amino acid chain, which is subsequently evaluated using the chosen empirical or synthetic landscape. For Fig 6D-6F this process is extended to the full set of 4^{3N} nucleotide genotypes. A critical detail in this process is the treatment of start and stop codons. For this study, we assume a protein becomes completely non-functional if a start or stop codon is modified within its sequence, and assign it the minimum observed fitness. Notably, this mapping does not itself alter the mutation kernel. Instead, it creates a class of landscapes with an inherent constraint: nucleotide configurations that encode the same protein must share identical fitness values. Because our analysis makes no prior assumptions regarding the fitness values within a landscape, it remains applicable to these models.

We considered three nucleotide mutation kernels. The uniform kernel assigns equal probability to each of the three possible substitutions. The directed kernels use species-specific nucleotide transition probabilities for *E. coli* and *A. thaliana*, derived from [41]. For each species, we also constructed a reversible weighted kernel. The directed matrix $\bar{D}$ was first symmetrised and then diag-

onally scaled,

$$\tilde{D} = Q \left(\frac{\bar{D} + \bar{D}^{\mathrm{T}}}{2} \right) Q, \tag{S3}$$

where Q was obtained by symmetric Sinkhorn scaling so that $\tilde{D}$ is symmetric and doubly stochastic. Thus, the weighted kernel retains non-uniform substitution weights while having a uniform stationary distribution. The original directed kernel remains row-stochastic and generally has a non-uniform stationary distribution.

Comparing the starting point sweeps from Fig 4D-4F and Fig 6D-6F, the global fits shown in Fig 6D-6F do not converge as closely to the analytical ρ_2 values as they do for landscapes without codon mapping. To investigate this further, we plot the true decay curves against the theoretical curves using analytical ρ_2 values in S3 Fig. The figure shows that the fitted ρ_2^{fit} values align much more closely with the true decay curves than the theoretical values. This discrepancy arises because the eigenvalues are significantly more dispersed in these landscapes, particularly after codon mapping. Increased eigenvalue dispersion degrades the fit of the analytical ρ_2, which approximates a weighted sum of exponentials by a single exponential. As Eq (14) shows, the analytical ρ_2 yields a decay curve that acts as a strict lower bound, as is visible in the figure.
