# Supplementary material for: Sequence-free landscape inference for directed evolution
Source: PLoS Comput Biol. 2026 Sep 3;22(9):e1014713. doi: 10.1371/journal.pcbi.1014713 (PMC13585340; doi:10.1371/journal.pcbi.1014713)
Supplement: Supplementary file 2 [file pcbi.1014713.s002.pdf]

S1 Table. Summary of ruggedness and decay-rate notation. Column F_μ denotes metrics computed from the average population fitness decay, whereas column G_μ denotes metrics computed from the squared average population fitness decay.

F_μ	G_μ	**Description**
ρ_1	ρ_2	Analytical decay rate.
ρ_1^{fit}	ρ_2^{fit}	Fitted decay rate.
	ρ_2^{loc}	Local fitted decay rate.
	ρ_2^{glob}	Global fitted decay rate.
ρ_{NK}		Closed-form NK ruggedness value.
ρ_{HoC}		Closed-form HoC ruggedness value.
	$\tilde{\rho}_2$	Decay rate for weighted, undirected mutation operator.
	$\bar{\rho}_2$	Decay rate for directed mutation operator.
