# Supplementary material for: Sequence-free landscape inference for directed evolution
Source: PLoS Comput Biol. 2026 Sep 3;22(9):e1014713. doi: 10.1371/journal.pcbi.1014713 (PMC13585340; doi:10.1371/journal.pcbi.1014713)
Supplement: Supplementary file 3 [file pcbi.1014713.s003.pdf]

S2 Table. Analytical decay rates under uniform, weighted, and directed mutation models computed on empirical landscapes.

Mutation model	GB1	TrpB	TEV	ParD3
Uniform (ρ_2)	0.4155	0.3034	0.2485	0.3380
$E. coli$ weighted ($\tilde{\rho}_2$)	0.4067	0.2811	0.2274	0.3134
Bound (17), $E. coli$ weighted	0.3512	0.3386	1.9027	0.5423
$E. coli$ directed ($\bar{\rho}_2$)	0.4040	0.2924	0.1764	0.3244
Bound (21), $E. coli$ directed	0.3545	0.3424	1.9923	0.5588
$A. thaliana$ weighted ($\tilde{\rho}_2$)	0.3988	0.2627	0.2103	0.2939
Bound (17), $A. thaliana$ weighted	0.6341	0.6114	3.4360	0.9793
$A. thaliana$ directed ($\bar{\rho}_2$)	0.3978	0.2677	0.1850	0.2991
Bound (21), $A. thaliana$ directed	0.6208	0.5981	3.4017	0.9646
