# Supplementary material for: Sequence-free landscape inference for directed evolution
Source: PLoS Comput Biol. 2026 Sep 3;22(9):e1014713. doi: 10.1371/journal.pcbi.1014713 (PMC13585340; doi:10.1371/journal.pcbi.1014713)
Supplement: Supplementary file 4 [file pcbi.1014713.s004.pdf]

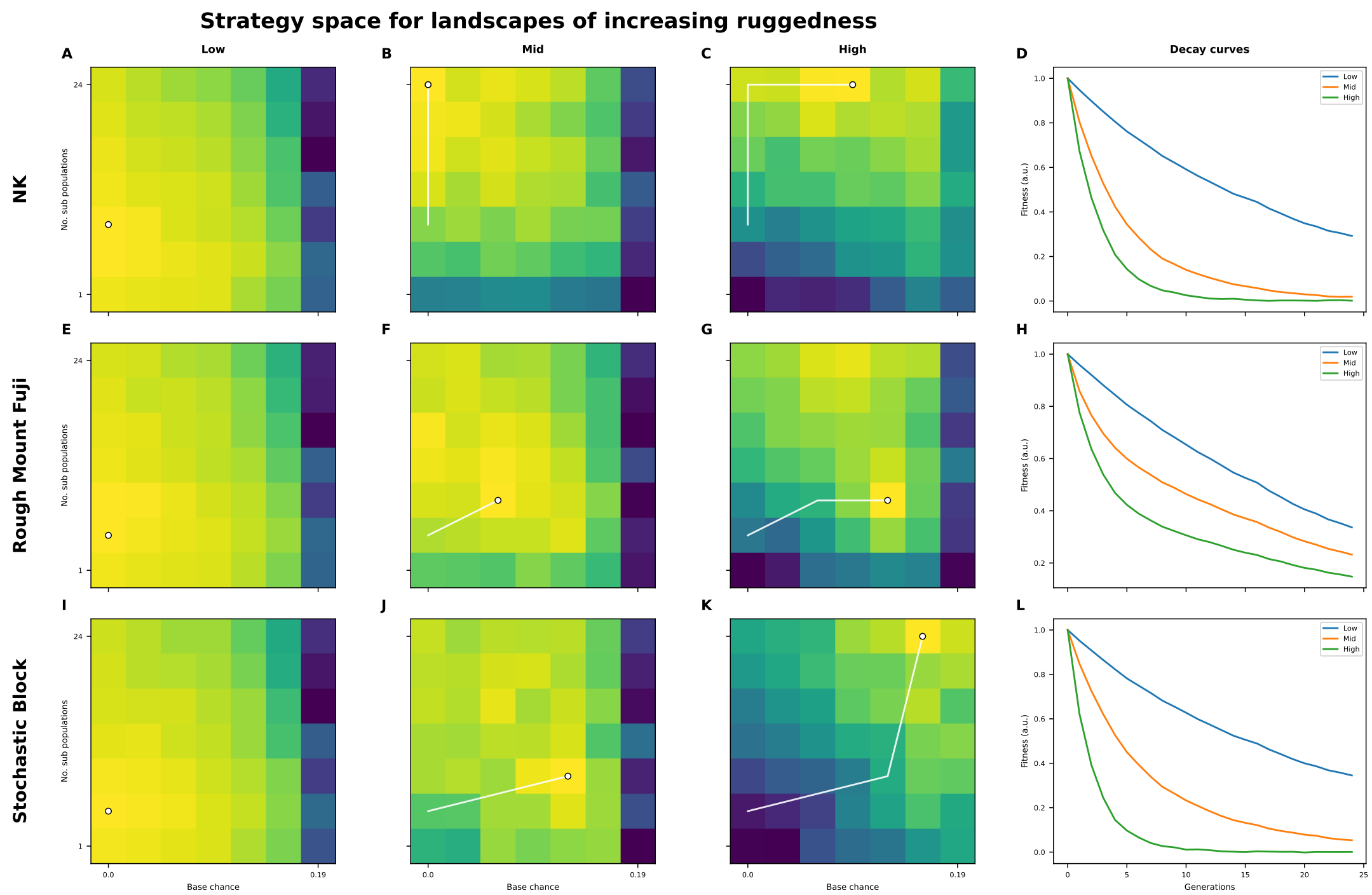


**S1 Fig. Comparison of strategy space and decay curve trends across different landscape models**. Heat maps show strategy spaces across low-, intermediate-, and high-ruggedness implementations of the $NK$, Rough Mount Fuji and stochastic block models. The white line tracks the optimal strategy as ruggedness increases. Each heat map represents an average across 300 starting points. Line plots on the right-hand side display the average fitness decay over the same landscapes represented in the heat maps.
