# Supplementary material for: Sequence-free landscape inference for directed evolution
Source: PLoS Comput Biol. 2026 Sep 3;22(9):e1014713. doi: 10.1371/journal.pcbi.1014713 (PMC13585340; doi:10.1371/journal.pcbi.1014713)
Supplement: Supplementary file 5 [file pcbi.1014713.s005.pdf]

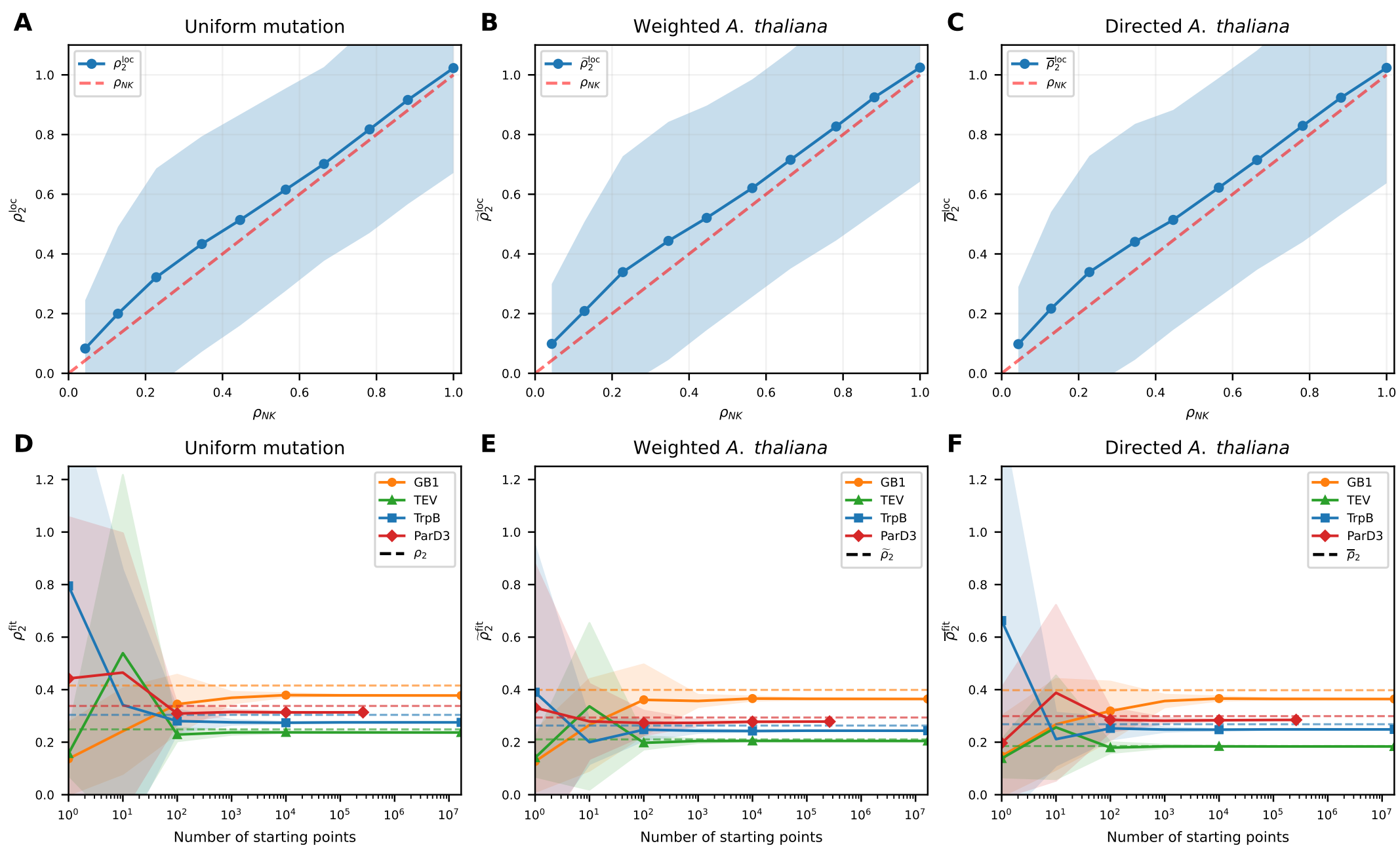

S2 Fig. *A. thaliana* mutation-model version of Fig 6A-6F. A–C: Local squared-fitness decay rates inferred from synthetic NK landscapes under uniform mutation (ρ_2^{loc}), weighted *A. thaliana* mutation ($\tilde{\rho}_2^{\text{loc}}$), and directed *A. thaliana* mutation ($\bar{\rho}_2^{\text{loc}}$). Points show binned means across local starts and shaded regions show standard deviations. The dashed red line marks ρ_{NK}. **D–F:** Full-nucleotide empirical landscape estimates for GB1, TEV, TrpB, and ParD3 as a function of the number of starting points. Dashed horizontal lines show the analytical ρ_2 for each landscape and mutation model.
