# Supplementary material for: Sequence-free landscape inference for directed evolution
Source: PLoS Comput Biol. 2026 Sep 3;22(9):e1014713. doi: 10.1371/journal.pcbi.1014713 (PMC13585340; doi:10.1371/journal.pcbi.1014713)
Supplement: Supplementary file 6 [file pcbi.1014713.s006.pdf]

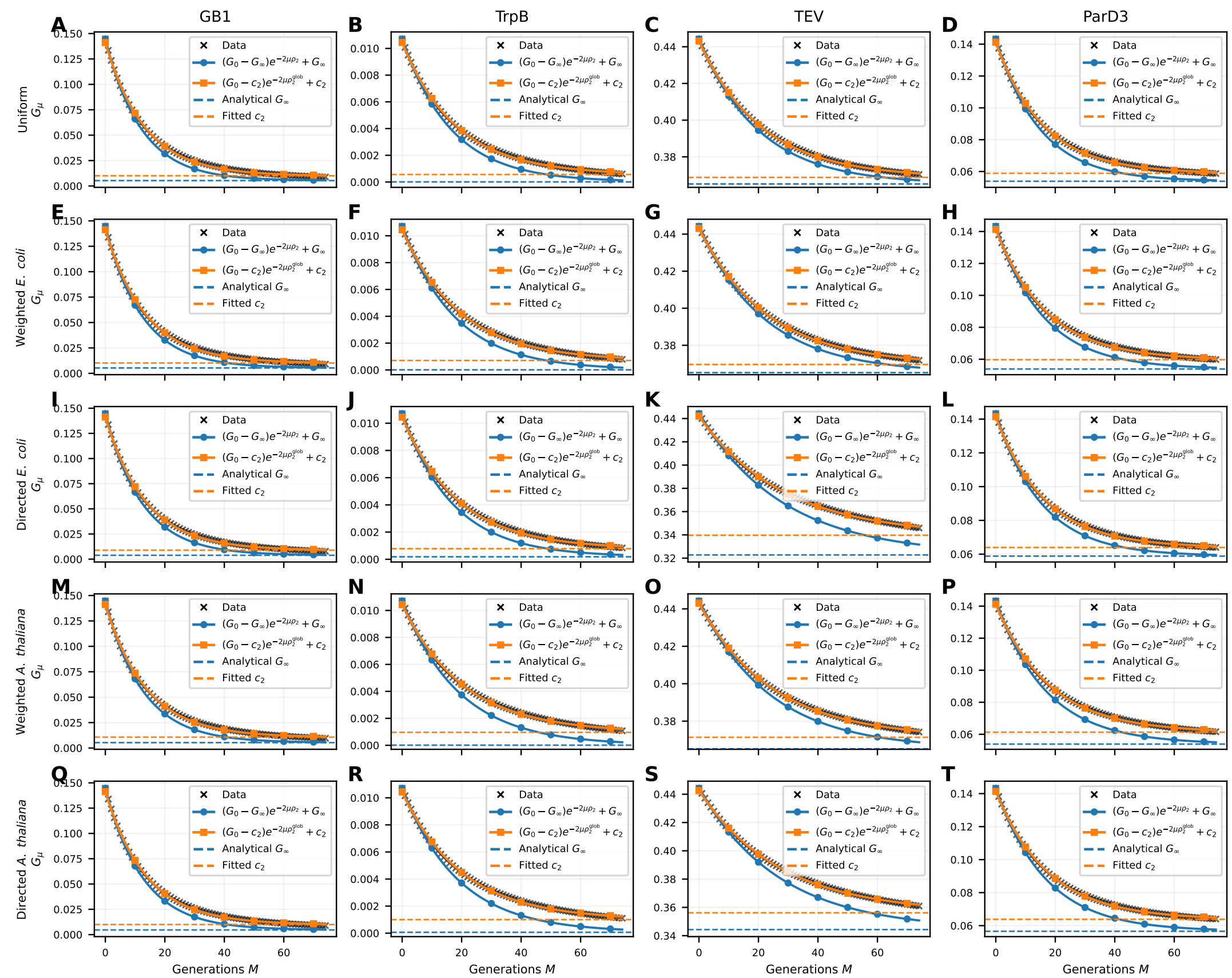

S3 Fig. Decay curves on empirical landscapes. Panels show full-nucleotide squared-fitness decay curves sampled from empirical landscapes, fitted decay curves using ρ_2^{glob}, and idealised analytical decay curves, which are computed as $(G_0 - G_\infty)\mathrm{e}^{-2\rho_2\mu} + G_\infty$. For uniform and weighted symmetric mutation models, G_∞ is computed from the unweighted landscape average. For directed asymmetric mutation models, G_∞ is computed using the stationary distribution of the nucleotide transition matrix. The mutation rate per generation is $\theta = 0.1/N$.
