# Supplementary material for: Sequence-free landscape inference for directed evolution
Source: PLoS Comput Biol. 2026 Sep 3;22(9):e1014713. doi: 10.1371/journal.pcbi.1014713 (PMC13585340; doi:10.1371/journal.pcbi.1014713)
Supplement: Supplementary file 7 [file pcbi.1014713.s007.pdf]

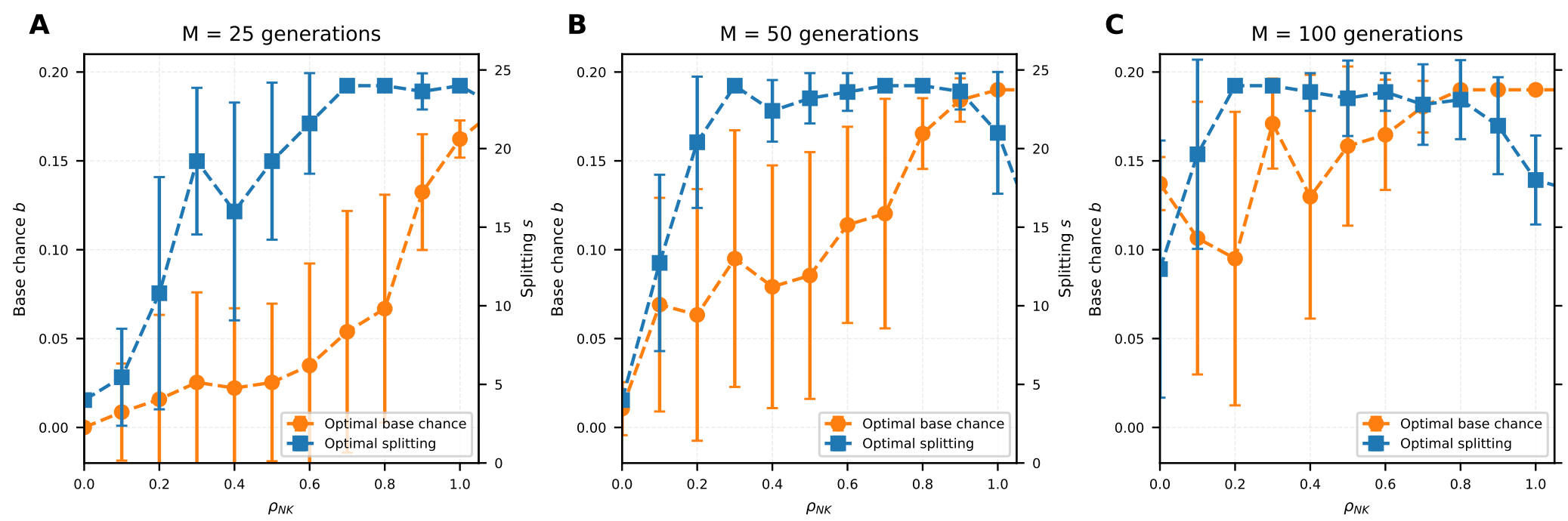

S4 Fig. Look-up table for NK landscapes with higher numbers of DE iterations. Parameters are as in Fig 5A, but with **A:** $M = 25$, **B:** $M = 50$ and **C:** $M = 100$ generations. Landscapes used a binary alphabet ($A = 2$), population size 1,200, mutation rate $\theta = 0.1$, and a 7×7 strategy grid over base chance $b \in \{0, 0.0317, 0.0633, 0.095, 0.1267, 0.1583, 0.19\}$ and splitting $s \in \{24, 20, 16, 12, 8, 4, 1\}$. The look-up was computed for 100 (N, K) pairs: $N \in \{10, 14, 18, 23, 27, 32, 36, 41, 45, 50\}$ with 10 sampled K values spanning low to high ruggedness for each N. For each (N, K) pair and DE horizon, the optimal strategy was selected after averaging over 25 replicate estimates per strategy point; the $M = 25$ grid used 200 random landscapes per (N, K) pair, while the $M = 50$ and $M = 100$ grids used 50. Points show the mean optimal parameter within each rounded $\rho_{NK} = (K + 1)/N$ bin, and error bars show the standard deviation of the optimal parameters across (N, K) pairs in that bin.
